# SCOTTI: Efficient Reconstruction of Transmission within Outbreaks with the Structured Coalescent

**DOI:** 10.1371/journal.pcbi.1005130

**Published:** 2016-09-28

**Authors:** Nicola De Maio, Chieh-Hsi Wu, Daniel J Wilson

**Affiliations:** 1 Institute for Emerging Infections, Oxford Martin School, University of Oxford, Oxford, United Kingdom; 2 Nuffield Department of Medicine, University of Oxford, Oxford, United Kingdom; 3 Wellcome Trust Centre for Human Genetics, University of Oxford, Oxford, United Kingdom; Duke University, UNITED STATES

## Abstract

Exploiting pathogen genomes to reconstruct transmission represents a powerful tool in the fight against infectious disease. However, their interpretation rests on a number of simplifying assumptions that regularly ignore important complexities of real data, in particular within-host evolution and non-sampled patients. Here we propose a new approach to transmission inference called SCOTTI (Structured COalescent Transmission Tree Inference). This method is based on a statistical framework that models each host as a distinct population, and transmissions between hosts as migration events. Our computationally efficient implementation of this model enables the inference of host-to-host transmission while accommodating within-host evolution and non-sampled hosts. SCOTTI is distributed as an open source package for the phylogenetic software BEAST2. We show that SCOTTI can generally infer transmission events even in the presence of considerable within-host variation, can account for the uncertainty associated with the possible presence of non-sampled hosts, and can efficiently use data from multiple samples of the same host, although there is some reduction in accuracy when samples are collected very close to the infection time. We illustrate the features of our approach by investigating transmission from genetic and epidemiological data in a Foot and Mouth Disease Virus (FMDV) veterinary outbreak in England and a *Klebsiella pneumoniae* outbreak in a Nepali neonatal unit. Transmission histories inferred with SCOTTI will be important in devising effective measures to prevent and halt transmission.

## Author Summary

We present a new tool, SCOTTI, to efficiently reconstruct transmission events within outbreaks. Our approach combines genetic information from infection samples with epidemiological information of patient exposure to infection. While epidemiological information has been traditionally used to understand who infected whom in an outbreak, detailed genetic information is increasingly becoming available with the steady progress of sequencing technologies. However, many complications, if unaccounted for, can affect the accuracy with which the transmission history is reconstructed. SCOTTI efficiently accounts for several complications, in particular within-patient genetic variation of the infectious organism, and non-sampled patients (such as asymptomatic patients). Thanks to these features, SCOTTI provides accurate reconstructions of transmission in complex scenarios, which will be important in finding and limiting the sources and routes of transmission, preventing the spread of infectious disease.

## Introduction

Understanding the dynamics of transmission is fundamental for devising effective policies and practical measures that limit the spread of infectious diseases. In recent years, the introduction of affordable whole genome sequencing has provided unprecedented detail on the relatedness of pathogen samples [1–4]. As a result, inferring transmission between hosts with accuracy is becoming more and more feasible. However, this requires robust, and computationally efficient methods to infer past transmission events using genetic information. Many complications, such as within-host pathogen genetic variation and non-sampled hosts, obscure the relationship between pathogen phylogenies and the history of transmission events, affecting the accuracy of such methods. Here, we present a new approach, SCOTTI, that accounts for these complexities in a computationally feasible manner.

**Fig 1:**
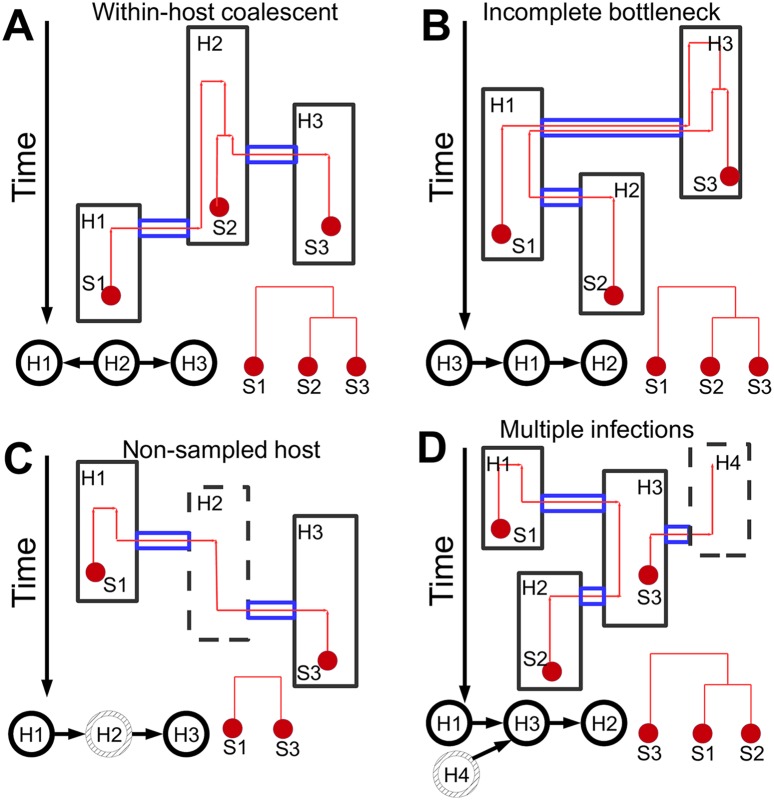
Examples of transmission complexities. Reconstruction of transmission can be hindered by several complexities causing disagreement between the actual transmission history and the phylogeny of the sampled pathogen. Here we show four examples of these complexities: **A**) Within-host evolution (similar to incomplete lineage sorting, can happen even with strong transmission bottlenecks), **B**) Incomplete transmission bottlenecks (or large transmission inocula) and within-host evolution, **C**) Non-sampled hosts (such as unknown or asymptomatic hosts), **D**) Multiple infections of the same host (or mixed infections). Different hosts (named H1, H2, and H3) are represented as black rectangles, and the rectangle with a dashed border represents a non-sampled host (a host for which no pathogen sample has been collected and sequenced, and for which there is no exposure time information). The top and bottom edge of each rectangle indicate the introduction and removal times, that is, the beginning and the end of the time interval within which a host is either infective or can be infected (e.g., arrival and departure time from the contaminated ward). Red dots represent pathogen sequence samples (respectively S1, S2, and S3), and red lines are lineages of the pathogen phylogeny. Blue tubes represent transmission/bottleneck events, where the contained lineages are transferred between hosts. Below each “nested” tree plot (representing phylogeny and transmission tree simultaneously, see Fig A in S1 Text), the corresponding transmission history is represented with black “beanbags”, and, in red, the phylogenetic tree of the sequences.

A number of approaches have been developed that reconstruct transmission from genetic data. One method, based on pathogen genetic data, rules out direct transmission if isolates from different hosts are separated by a number of substitutions above a fixed threshold [5–7]. This approach cannot generally distinguish direct transmission from transmission through one or more intermediate hosts, or infer its direction. Alternatively, the phylogenetic tree of the pathogen samples is often used as a proxy for the transmission history [8, 9]. While phylogenetic signal can be very informative of transmission, it can also be misleading [10, 11]. The main cause of this problem is within-host variation that can generate discrepancies between the phylogenetic and epidemiological relatedness of hosts, and can bias estimates of infection times [12]. One problem arising from within-host diversity is that the pathogen isolates transmitted by a host are not necessarily genetically identical to those sampled from the same host. This phenomenon can be mathematically modelled using population genetics approaches such as the coalescent [13], to describe within-host evolution (Fig 1A). Other factors that can cause disagreement between phylogeny and transmission history are: (i) Incomplete transmission bottlenecks, where some of the within-host genetic variation is transmitted from donor to recipient through a non-negligibly small inoculum; this means lineages from the same host may not have shared a common ancestor since long before the time of infection of the host (Fig 1B). (ii) Non-sampled hosts, such that a sampled patient is not necessarily linked by direct transmission to its most closely related sampled patient, but can have a non-sampled intermediate (Fig 1C) [14]. (iii) Multiply infected hosts, that can cause patients to be erroneously excluded from some transmission chains, in particular if multiple samples from the same patient are not collected (Fig 1D).

Several methods emerged in recent years explicitly modelling both the transmission process and genetic evolution to perform inference of the history of transmission events [11, 14–26]. These methods generally make use of epidemiological dating information (such as the date of sampling, the interval of exposure of a host to an outbreak, or the likely duration of infectiousness), but they usually ignore within-host variation and other causes of phylogenetic discordance with transmission history [14–20, 22, 23]. The methods of Ypma and colleagues [21], Didelot and colleagues [24], and Hall and colleagues [25] account for within-host diversity, but assume that all hosts in the outbreak have been detected and sequenced, which may be incorrect or uncertain in practical settings.

Here, we propose a new Bayesian approach called SCOTTI (Structured COalescent Transmission Tree Inference) that not only accounts for diversity and evolution within a host, but also for other sources of bias, namely non-sampled hosts and multiple infections of the same host. This new method builds on our recent progress in efficiently modelling migration between populations using an approximation to the structured coalescent [27]. Formally, we model each host as a separate pathogen population, and we model transmission as migration between hosts. SCOTTI has a broad range of applicability as it relaxes the typical assumptions that every host is sampled and that there is no within-host variation (see Fig B in S1 Text). A limitation of our method is that we do not model transmission bottlenecks. This can be a disadvantage with strong bottlenecks at transmission (due to small inocula), but on the other hand it may be an advantage with large transmission inocula. SCOTTI is implemented as an open-source package for the Bayesian phylogenetic software BEAST2 [28], and as such, it can be freely installed and used. We compare the performance of SCOTTI and the popular software Outbreaker [22] (version 1.1-5) on simulated data and on real datasets of Foot and Mouth Disease Virus (FMDV [29]) and *Klebsiella pneumoniae* [30]. These applications highlight how the two methods usually provide very different interpretations of outbreak dynamics, with SCOTTI showing typically higher accuracy on simulated data. By combining epidemiological and genetic information, and by implementing a general and computationally efficient model, SCOTTI can accurately infer transmission in a broad range of settings, providing important information to understand and limit the spread of infectious disease.

## Results

### SCOTTI: A New Approach to Reconstructing Transmission Events

**Fig 2:**
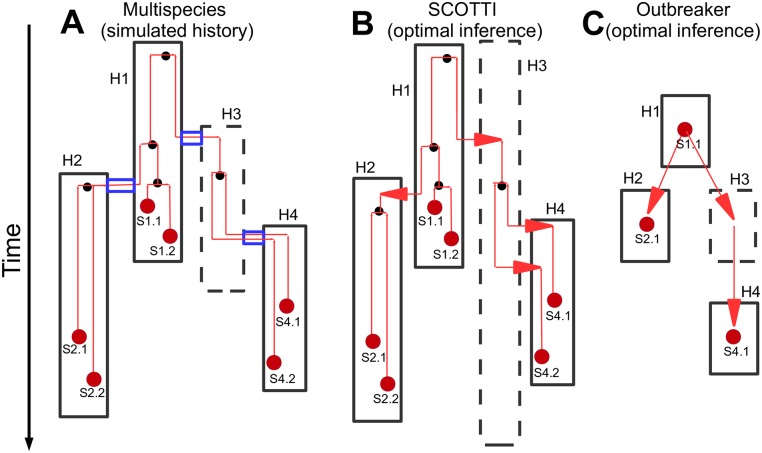
Graphical representation of models of transmission and evolution. In the present work we consider three different models of pathogen evolution within an outbreak: **A)** The multispecies coalescent model with transmission bottlenecks, used for simulations, **B)** The structured coalescent (SCOTTI) model used for inference, **C)** The Outbreaker model also used for inference. The pictures highlight some key parameters and features of the models. Different hosts (H1, H2, H3, and H4) are represented as black rectangles. The top and bottom edge of each rectangle are the introduction and removal times of the respective hosts in **A** and **B**. The hosts with a dashed border are non-sampled. Red dots represent samples (only one per host allowed by Outbreaker), red vertical lines are lineages of the phylogeny. Smaller black dots represent coalescent events. Red arrows are transmissions/migrations in **B** and **C**. Blue tubes are transmissions with bottlenecks in **A**, and transmitted lineages are contained within them. In **A**, a transmission bottleneck from host H1 to H2 causes two lineages in H2 to coalesce (find a common ancestor backwards in time) at the same time of transmission. This does not happen at the transmission from H3 to H4, where the two lineages in H4 do not coalesce (incomplete bottleneck) and are both inherited from H3 to H4 at a single transmission event.

Many methods that infer transmission from pathogen genetic data assume that the pathogen population within a host is genetically homogeneous, thereby overlooking within-host variation. A popular example is Outbreaker [22], where pathogen genetic mutations are assumed to happen during transmission between hosts, and not within hosts (Fig 2C). The simplicity of this model allows estimation of transmission events for pathogens with short and regular incubation and recovery times, and with negligible within-host variation. However, within-host evolution and overlapping infection intervals are often not negligible for most pathogens, particularly for bacterial infections and chronic viral infections. If unaccounted for, these complexities can lead to misleading inference concerning transmission events [11, 12].

A natural way to account for within-host evolution is via an extension of the multi-species coalescent model [31] including transmission bottlenecks. In this model, each host constitutes a separate pathogen population, and with a transmission event some isolates are transmitted to, and colonize, a new host, instantaneously growing to a full and constant-size pathogen population (see e.g. [24, 32, 33] and Fig 2A). While this multispecies model is advantageous in several respects, it is often too computationally demanding to be useful for inference. In fact, implementations of the multispecies coalescent approach typically parametrize the transmission history (who infected whom and when); in cases when there is a strong uncertainty over the transmission history, for example in the presence of many non-observed hosts and considerable within-host variation, this leads to a significant computational burden. So while we base SCOTTI on a simplified model, we simulate pathogen evolution (to assess and compare methods performance) under the multispecies model. In these simulations, we fix the host-to-host transmission history, and simulate evolution of pathogen lineages within hosts. We use two distinct transmission histories taken from the literature [8, 15]. We simulate under a broad range of scenarios: different transmission bottleneck severities (weak vs. strong), one vs. two samples per host, different numbers of non-sampled hosts, different amounts of genetic information, and different times of sampling (early, vs. late, vs. randomly within a host infection). We give further details on the simulation scenarios in the Materials and Methods.

While we simulate outbreak data under the multispecies model, to infer transmission we propose a model based on the structured coalescent, SCOTTI. In the structured coalescent multiple distinct populations are present at the same time, lineages in the same population can coalesce (find a common ancestor), and lineages can migrate between populations at certain rates. In SCOTTI, each host represents a distinct pathogen population, and migration of a lineage represents a transmission event (Fig 2B). Lineages are only allowed to evolve within, and migrate to, hosts that are exposed at a given time, and exposure times are informed by epidemiological data. In SCOTTI, different lineages within the same host can migrate, backward in time, into different hosts at different times; on the other hand, in the multispecies coalescent model all extant lineages within a host have to move together (again backward in time) into the donor host at a single point in time, so that each host can only be infected once. Under our new model, we perform estimation using a new implementation of BASTA (BAyesian STructured coalescent Approximation), an efficient approximation to the structured coalescent [27], adapted to this epidemiological setting. The use of the approximations in BASTA substantially reduces computational demand, in particular when many populations are considered. In all cases of simulation and inference, we do not model selection, but assume neutral genome evolution. More details on SCOTTI are provided in the Materials and Methods.

### Accuracy of Inference on Simulated Data

**Fig 3:**
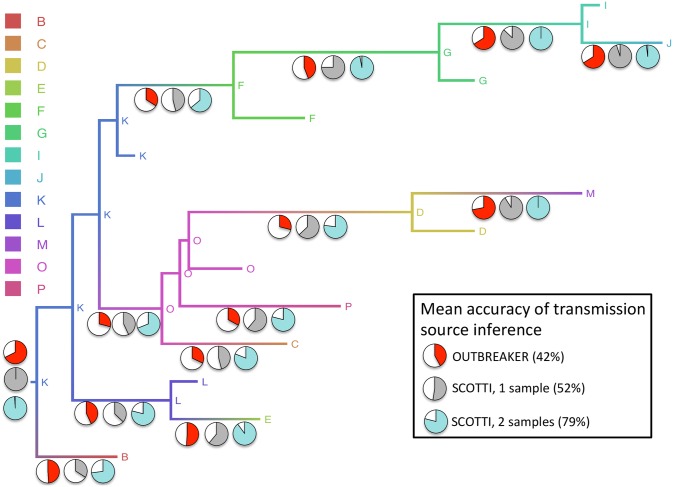
Accuracy of SCOTTI vs. Outbreaker in the base simulation scenario. In our base simulation setting, SCOTTI has higher accuracy than Outbreaker, in particular when provided multiple samples per host. The coloured “Maypole” tree (see Fig A in S1 Text) represents the first transmission history used for simulations, with one colour associated to each host, internal nodes corresponding to infection events and times, and tips representing infection clearance times. The pie charts refer to the accuracy of transmission estimation in the base scenario with strong bottleneck. The coloured slice in each pie chart is the proportion of replicates (out of a total of 100) for which the correct origin of transmission has been correctly inferred. Pie charts are plotted below the branch corresponding to the transmission they refer to, while the pie charts for the index host K are plotted next to the root.

**Fig 4:**
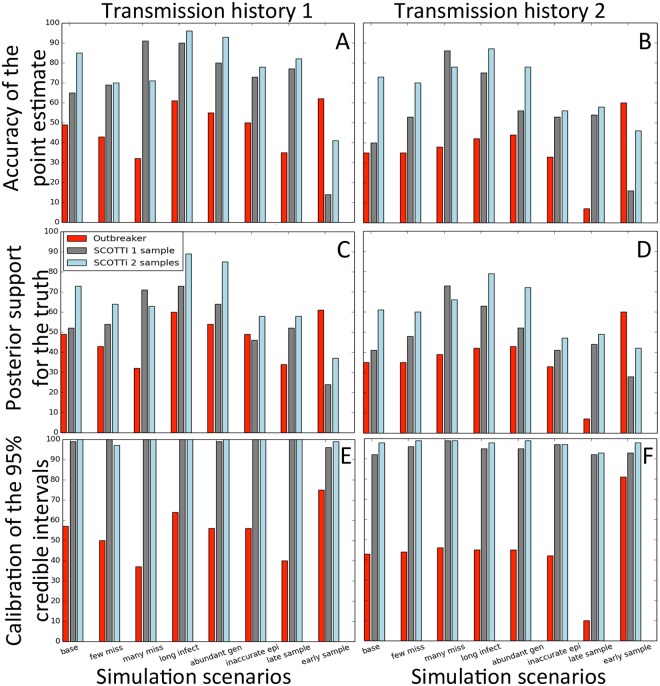
Summary of transmission inference accuracy. SCOTTI shows higher accuracy than Outbreaker in all scenarios except with early sampling, while Outbreaker credible sets are poorly calibrated. Pathogen sequence evolution was simulated under transmission history 1, used in **A** and **C**, and transmission history 2, used in **B** and **D**. In **A** and **B** bars represent proportions, expressed as percentages, of correct inferences of transmission origin (i.e. donor host) over 100 replicates and all transmission events for each method (differentiated by colour as in legend). On the X axis are different simulation scenarios. In **C** and **D** bars represent average posterior supports, again expressed as percentages, for the correct sources over all patients and replicates. In **E** and **F** bars represent proportions (expressed as percentages) of 95% posterior credible sets that contain the simulated (true) origin. The 95% posterior credible set for a host is the minimum set of origins with cumulative probability ≥95%, and such that all origins in the set have higher posterior probability than all origins outside of it.

To test the accuracy of our new method SCOTTI in inferring the origin of transmission, and to compare it to the accuracy of the software Outbreaker, we simulated pathogen evolution within two distinct, fixed transmission histories, one from a 2001 FMDV outbreak [15] and one from an HIV outbreak [8]). We measure the accuracy of the method as the frequency of correct point estimates of transmission source. This source can be either a specific sampled (and observed) host, or a generic non-sampled host. As the point estimate we take the transmission donor with the highest posterior probability. In the base simulation scenario (random sampling times, low genetic variation, every host sampled) SCOTTI performs well in the first transmission history (65% and 85% mean accuracy with respectively one or two samples per host), and less well on the second transmission history (40% and 73% accuracy, see Fig 3 and Fig C in S1 Text). In fact, while in the first transmission history transmission events are quite homogeneously distributed through time, in the second history they happen very early, with samples collected much later. Bottleneck size seems to have a limited effect on inference, although we did not simulate extremely weak bottleneck. Looking at all other simulation scenarios, we observe that the accuracy of SCOTTI remains consistently high, with the noticeable exception of the case in which sampling occurs very early in infection (Fig 4). One likely reason for this is that SCOTTI does not model transmission bottlenecks. With early sampling, coalescent events are likely to happen in the limited time interval between infection (corresponding to the transmission bottlenecks) and sampling. Because it is not constrained, SCOTTI often places such coalescent events in the wrong host.

Overall, SCOTTI shows higher accuracy than Outbreaker across scenarios (Fig 4). Generally Outbreaker has poor accuracy in estimating the source of transmission for most links in our base scenario, with an average of 49% in transmission history 1 and 35% in transmission history 2 (Fig 3 and Fig C in S1 Text). Its limited performance can be largely explained by the fact that Outbreaker does not account for within-host variation. This showcases the utility of an approach of broader applicability such as SCOTTI. SCOTTI generally outperforms Outbreaker even with a single sample per host, and as more samples are included, it achieves greater accuracy (85% and 73% mean accuracy, vs. 49% and 35% of Outbreaker). The only instance of inaccuracy of SCOTTI is the transmission from host P5 to host P6 (Fig C in S1 Text), probably due to the limited phylogenetic evidence supporting it. Outbreaker, on the other hand, shows acceptable accuracy when the order of transmission is largely reflected by the order of sampling, and low accuracy otherwise, as for example for the transmissions from host P1 to host P8 and from host P1 to host P5 (Fig C in S1 Text).

Another difference between the two methods is that Outbreaker tends to infer a posterior distribution supporting a narrower range of origins. This, paired with its limited accuracy, leads the method to exclude a true origin from 95% credible sets in about half of the simulations. SCOTTI is instead much better calibrated, with 95% credible sets containing the true origin between 90% and 100% of the time (Fig 4E and 4F). Also, most of the inaccurate inference of SCOTTI derives from assigning the source of transmission to non-sampled hosts, i.e., SCOTTI is tentative in naming sampled hosts as transmission donors. While it is possible to inform SCOTTI of the absence of non-sampled hosts by specifying a strong prior on the corresponding parameter, we prefer not to do so, since in general this information is not available for real outbreaks. In Outbreaker, inference errors often involve misattributing the source of infection source to one of the sampled hosts (Fig D in S1 Text). This considerably affects estimation when genetic and epidemiological data from some hosts is withheld (one host in the “few missing” scenario and three hosts in the “many missing” scenario). In these settings the accuracy of SCOTTI often increases (as non-sampled hosts are correctly attributed to be the source), while it decreases for Oubreaker (since infection source is wrongly attributed to sampled hosts, see Fig E in S1 Text).

In most of our scenarios the amount of genetic information available to distinguish different transmission histories is rather limited, with 2-3 SNPs per sampled host (total number of SNPs divided by the number of sampled hosts) on average. From such data a single phylogenetic tree relating the sequenced samples cannot be inferred unambiguously. When we increase phylogenetic signal, either by simulating longer infection times (“long infection” scenario) or with longer genetic sequences (“abundant genetic”), the accuracy of the methods substantially increases (Fig F in S1 Text).

SCOTTI and Outbreaker require distinct formats for epidemiological information. Outbreaker requires as input a probability distribution over the possible durations and intensity of infectivity, and sampling times. In contrast, SCOTTI requires the user to specify an exposure interval for each host. In simulations where the exposure intervals provided for each host were doubled in length compared to the true ones (“inaccurate epi” scenario) SCOTTI appeared relatively robust (Fig 4 and Fig G in S1 Text).

Furthermore, we investigated the effect of sampling times on the two methods. Outbreaker has higher accuracy when sampling times are close to the start of infection (“early sampling” scenario). Indeed this is the one setting in which Outbreaker outperforms SCOTTI in inference accuracy (Fig 4). In this case SCOTTI is overly tentative in identifying sampled hosts as the source of transmission, and is overly conservative in its quantification of uncertainty (as in the second transmission tree with two samples per host, Fig D in S1 Text). A contributory factor to this behaviour is that SCOTTI does not model transmission bottlenecks, and so a sampled lineage is not readily inferred to have coalesced (to have found a common ancestor with another lineage) in the short time between sampling and infection, and therefore infers too great a contribution of non-sampled hosts. In situations where a single host transmits multiple times in close succession (as patient P1 in the second transmission history), lineages from its recipients will tend to coalesce within P1 in random order before coalescing with the samples from P1. This means that phylogenetic trees are relatively uninformative of the transmission history, and this inflates uncertainty in the identification of the transmission source. In contrast, Outbreaker is less accurate when sampling times are close to clearance times (“late sampling” scenario), see Fig H in S1 Text.

We did additional simulations to test the performance of SCOTTI under random transmission histories, variable host features, different levels of within-host genetic variation, and proportions of non-sampled hosts (see Materials and Methods). The overall performance of SCOTTI on random transmission histories is in between those observed in the two real transmission histories: between 50 and 60% of accuracy of the point estimates, and calibration above 90% (Fig I in S1 Text). Proportion of non-observed cases, variable within-host effective population size, and variable host infectivity, seem all to have little effect on the accuracy and calibration of SCOTTI. An increase in proportion of non-sampled hosts can even lead in an increase of accuracy; in fact, this leads to a higher proportion of sampled hosts infected by non-sampled hosts. At the same time, the epidemiological distance between sampled hosts increases, and so it becomes easier for SCOTTI to exclude direct transmission between sampled hosts. On the other hand, as we increase the within-host genetic variability by reducing the effect of the transmission bottleneck and increasing the within-host effective population size, we notice that the accuracy of the point estimate of SCOTTI goes remarkably down, while calibration remains at acceptable levels (Fig J in S1 Text). However, providing two samples per host increases the accuracy, supporting the idea that, if available, many sequences, or deep sequencing, from each host could provide sufficient information even with inherently difficult scenarios [26].

We simulated a number of outbreaks of varying number of hosts and samples to test the computational applicability and efficiency of SCOTTI (see Materials and Methods). These simulations show that the number of hosts infected at any time is a more important determinant of the computational cost of SCOTTI than the length of the outbreak (Fig K in S1 Text). Also, SCOTTI can investigate a dataset of 50 hosts and 2 samples per host in 1-2 hrs using a single processor.

### Analysis of FMDV and *Klebsiella pneumoniae* Outbreaks

To investigate the impact of our method on the study of real outbreaks, we examined the transmissions inferred by SCOTTI and Outbreaker in two real outbreaks of FMDV in 2007 [29] and *K. pneumoniae* in 2011-2012 [30].

**Fig 5:**
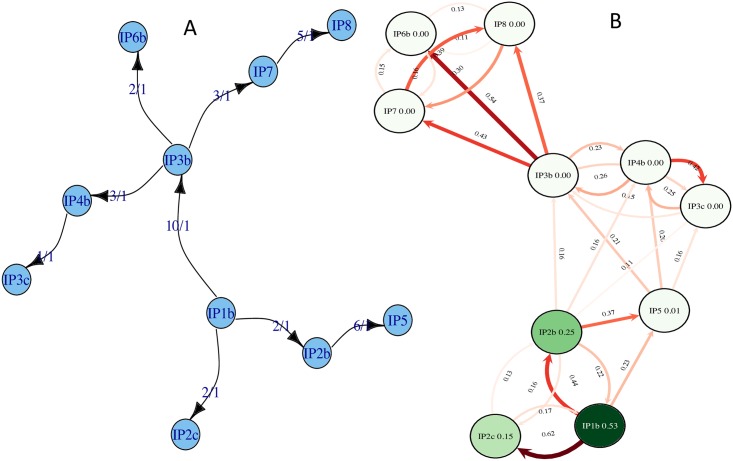
Reconstruction of transmission events in a FMDV outbreak. Outbreaker (**A**) and SCOTTI (**B**) provide different interpretations of the 2007 South of England FMDV outbreak. **A)** “Beanbag” tree (see Fig A in S1 Text) of Transmission events inferred with Outbreaker. The two numbers on each transmission arrow represent respectively the number of nucleotide substitutions separating two hosts, and the inferred posterior support of the event (in this case always 1, meaning 100% support). All transmissions are inferred to be direct with more than 95% posterior probability. **B)** “Beanbag” tree of transmission events inferred with SCOTTI. Numbers within host circles represent the posterior probabilities of the corresponding host being the index host (the root) of the considered outbreak. Numbers on arrows represent the inferred posterior probabilities of the corresponding direct transmission events. Colour intensity is proportional to posterior probability.

FMDV infects cloven-hoofed animals, and is an economically devastating disease for the farming sector. The 2007 FMDV outbreak occurred in the South England as two distinct transmission clusters, one in August and one in September (Fig L in S1 Text), at an estimated cost to the economy of more than 100 million pounds [29]. Among the questions facing investigators were the source of the outbreak, and the connection between the August and September clusters. Following previous investigations of this outbreak, we studied transmission at the farm-to-farm level, rather than at the scale of individual animals, by taking farms or other geographically delimited premises as the unit of transmission. Based on previously published whole genome FMDV data [29], Outbreaker estimated transmission events with high certainty (100% posterior probability, Fig 5A). Yet, some of these inferred events are inconsistent with exposure data and with the transmission events inferred in [29] using genetic and epidemiological data (Fig L in S1 Text). For example, Outbreaker infers *IP*1*b* → *IP*3*b* and *IP*3*b* → *IP*4*b* instead of *IP*4*b* → *IP*3*b* and *IP*5 → *IP*4*b* [29]. This is in part because Outbreaker does not make use of host-specific exposure data, and that its model of genetic evolution does not account for the fact that a host sampling time can be distant from its infection time, as is probably the case for host IP5 here, which was possibly subject to infection for a longer time than other hosts. SCOTTI instead considers a much broader range of possible transmission events (Fig 5B). The transmission origins with highest posterior probability inferred by SCOTTI correspond to those inferred in [29], consistent with the fact that both methods use exposure data. Another reason to believe that SCOTTI is more reliable in this case is the sampling scheme, which is very close to our simulated “late sampling” scenario, where we observed SCOTTI to be more accurate (Fig 4). As shown in simulations, much of the uncertainty in SCOTTI is attributed to the possible presence of non-sampled hosts, which could be reduced by modifying the prior on the number of non-sampled hosts. Regarding the connection between the August and the September clusters, Cottam and colleagues identified IP5 as a possible link in the transmission chain, but did not exclude the possibility of alternative, transient, unobserved infections between the two clusters. SCOTTI adds weight to the notion of unobserved and non-sampled intermediate infections; the posterior probability of non-sampled intermediates was ≈ 66%, suggesting that it is likely that some important transmission links might not have been sampled.

We also investigated the same outbreak using Beastlier [25]. This method implements an exact variant of the multispecies coalescent model that we use in our simulations. However, so far (version of July 2016) it does not allow non-sampled non-observed hosts, so it cannot be used to address the issue of possible missing links. Unlike SCOTTI, Beastlier also forbids the specification of an earliest date of infection, and so we could only use a strict subset of the epidemiological information. We found that, similarly to SCOTTI, Beastlier shows a large degree of uncertainty in the inference of the transmission tree (Fig M in S1 Text). However, some of the transmission events inferred by Beastlier with high posterior probability are not consistent with SCOTTI or the full epidemiological data (*IP*1*b* → *IP*3*b*, *IP*3*b* → *IP*2*c* and *IP*6*b* → *IP*5).

**Fig 6:**
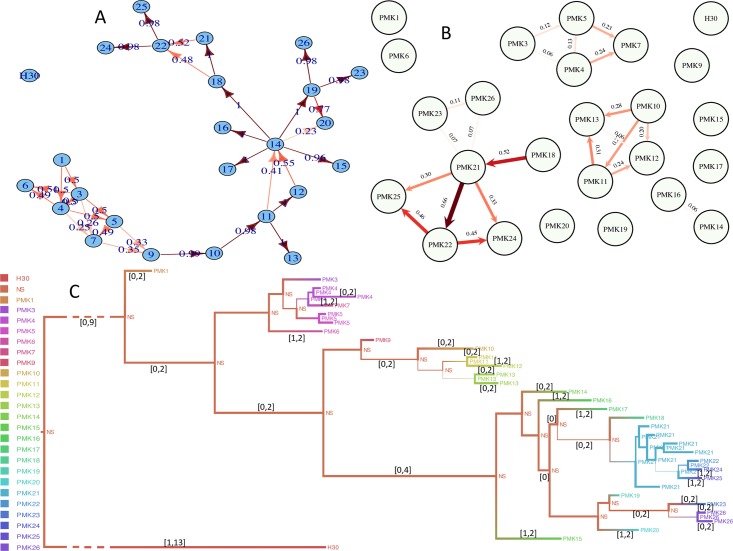
Reconstruction of Transmission events in a *K. pneumoniae* outbreak. Outbreaker (**A**) and SCOTTI (**B** and **C**) provide different interpretations of the *K. pneumoniae* outbreak. **A)** “Beanbag” tree of transmission events inferred with Outbreaker. Each circle represents a host, with “PMK” removed from their name. The number on transmission arrows represents the inferred posterior probability of the event. All arrows represent direct transmissions (without intermediate non-sampled hosts, with more than 85% support) except the one from PMK9 to PMK10 which is inferred to be through at least one intermediate host. **B)** “Beanbag” tree of transmission events inferred with SCOTTI. Numbers on arrows represent the inferred posterior probabilities of the corresponding direct transmission events. Colour intensity is proportional to posterior support. **C)** “Maypole” maximum clade credibility tree (see Fig A in S1 Text) inferred with SCOTTI, annotated and coloured with the highest posterior probability hosts for internal nodes. “NS” represents all non-sampled hosts. Branch width indicates the posterior probability of the inferred host at the node at the right end of the considered branch. Branches are annotated with 95% posterior intervals of the number of transmissions. For non-annotated branches, the interval is [0, 1].

As another example of empirical analysis, we also investigated an antimicrobial resistant *K. pneumoniae* outbreak in a Nepali neonatal intensive care unit between August 2011 and June 2012. *K. pneumoniae* antimicrobial resistant strains are a major health concern particularly for neonatal clinical care. In this outbreak, there were 16 neonate deaths out of 25 infections, representing a very high case fatality rate (64%) [30]. Of major importance to the outbreak investigation was the role of transmission within the unit, vs recurrent introduction from outside. In this outbreak, Outbreaker inferred just one transmission event to be indirect (through a non-sampled host, from host PMK9 to PMK10, with probability ≈ 91%). All other transmission events were inferred to be between sampled hosts with posterior probability above 85% (although this probability was often distributed over multiple possible sampled sources). Two patients were inferred to represent novel introductions (index cases): PMK1 and H30. While most infections were attributed to a single patient with greater than 99% probability, the source of infection of many patients was considerably uncertain (PMK3-9, PMK14, PMK20 and PMK22, see Fig 6A). SCOTTI, in complete contrast, inferred a non-sampled source as the most likely for the majority of sampled patients (Fig 6B). Direct transmission between sampled hosts was only inferred with high confidence for a small number of pairs, the most likely being *PMK*18 → *PMK*21, *PMK*22 → *PMK*24, *PMK*22 → *PMK*25. Overall, SCOTTI inferred sampled patients to constitute a small portion of the total outbreak, forming separated (Fig 6B), and yet related (Fig 6C), sub-outbreaks (respectively PMK3-7, PMK9-13, PMK14-26, and the two relatively isolated cases PMK1 and H30) within a larger outbreak. This is consistent with, and informed by, the presence of four time intervals not covered by any host exposure, requiring the presence of non-sampled infected intermediate hosts, recurrent introductions, or environmental contamination [30] (Fig N in S1 Text). SCOTTI also inferred most of the common ancestors of the sampled patients to be non-sampled (Fig 6C). This conclusion cannot be reached by Outbreaker, which assumes that the most recent common ancestor of two sampled cases (within outbreaks with a single index case) is also sampled.

## Discussion

Methods to infer transmission events within outbreaks are essential to determine the causes and patterns of transmission, and therefore to inform policies preventing and limiting transmission. Genomic data from pathogen samples give the opportunity to investigate, at an unprecedented level of detail, the relatedness of pathogens from different hosts. However, common real life complexities such as within-host variation (in particular for bacterial and chronic viral infections [10–12]), or hosts that have not been sampled (e.g. unknown or asymptomatic patients) can hinder the reconstruction of transmission events. Therefore, methods that efficiently infer transmission from genomic data while accounting for within-host variation and non-sampled hosts are essential if we want to determine specific transmission events within outbreaks, or even general patterns of transmission which might inform policies and recommendations for prevention of infection.

Here we have presented SCOTTI, a novel method of host-to-host transmission inference (who infected whom) that is built around a computationally efficient model of pathogen evolution based on the structured coalescent. By modelling each host as a distinct pathogen population, and transmission as migration of lineages between hosts, we have shown that it is possible to model within-host evolution and estimate transmission events with good accuracy, even in the presence of non-sampled hosts. We compared the accuracy of SCOTTI with that of the similar software Outbreaker [22] in a broad range of simulation scenarios: different epidemics (FMDV [15] or HIV [8]), transmission bottleneck sizes, numbers of samples per host, numbers of non-sampled hosts, genome sizes, SNPs per transmission, and sampling times. Overall, SCOTTI has better accuracy, in particular when benefitting from multiple samples from each host, which Outbreaker does not allow. SCOTTI, in fact, explicitly models within-host evolution, and multiple samples from the same host can be particularly informative regarding the within-host coalescent rate, and, in theory, the proportion of mixed infections. While it is common to sample and sequence only one haplotype from each host, this clearly shows that transmission inference benefits greatly from within-host variation data. Also, SCOTTI explicitly uses epidemiological data in the form of exposure times of the hosts to inform plausible direct transmission events, or equivalently to rule out impossible direct transmissions due to non-overlapping exposure times. Inaccurate epidemiological data, and even more so the absence of such data, can consequently lead to a decrease in accuracy. In our simulations, we have observed that partial inaccuracy in epidemiological data has a modest effect on the accuracy of SCOTTI; yet, we expect that in the complete absence of exposure time information, differently from Outbreaker, the accuracy of SCOTTI would be more deeply compromised. Further, SCOTTI explicitly models non-sampled hosts, and this helps inference when the outbreak is only partially sampled, as we show in simulations and in the *K. pneumoniae* outbreak. We showed in particular that not only do SCOTTI and Outbreaker have different accuracy, but also differ with respect to the inferred contribution of non-sampled hosts. It is therefore important to carefully select the most appropriate software to investigate transmission, and looking at the degree of concordance of different methods might reveal transmission events of difficult attribution.

Although SCOTTI has broad applicability, it has important limitations to be considered that we will address in future work. One problem is that SCOTTI ignores transmission bottlenecks, that is, the rapid growth in pathogen population size within a host following transmission. While the presence of strong transmission bottlenecks alone does not seem to cause an increase in error in SCOTTI, the presence of bottlenecks in conjunction with very early samples (close to the time of start of exposure) can considerably affect the accuracy of SCOTTI, as we showed in the simulation setting involving early sampling times. A somewhat ad-hoc solution to this problem could be to artificially shift back the starting time of exposure for hosts sampled very early, as we have also shown that such a decrease in informativity of epidemiological data has limited effect on SCOTTI. On the other hand, due to the absence of transmission bottlenecks in its model, SCOTTI is likely to suit outbreaks with frequent mixed infections and large transmission inocula (see e.g. [34]), like many gut and environmental bacteria which have high prevalence and low pathogenicity. The model could also be extended in the future to allow different types of epidemiological data (such as a prior on the time of each infection, while we now only allow a fixed older bound); compartmental epidemiological models [35], and geographic distance or structure of different hosts; or additional parameters describing host infection times that could increase the model realism while not necessarily reducing its computational performance (see e.g. [39]).

Transmission tree topology also seems to have an important effect on the observed patterns (see e.g. [36]), in particular we notice that in the presence of a super-spreader that infects multiple individuals in a short time span, as in Fig C(B) in S1 Text, it is inherently hard to distinguish different possible transmission routes because of the little discriminatory power provided by genetic and epidemiological data, and due to the randomness of the within-host coalescent process. However, by including information regarding the distribution of incubation times and the dates of occurrence of symptoms, when available, it should be possible to increase the accuracy of inference by further reducing the space of plausible transmission histories. While we have not investigated the effect of outbreak size on inference accuracy, in principle we expect two patterns: firstly, accuracy should decrease as the number simultaneous epidemiologically closely linked cases increases; in fact, the presence of multiple possible donors would increase uncertainty regarding the sources of infection. Secondly, longer outbreak should not have decreased accuracy, because cases distant in time cannot be linked; increased outbreak length could even increase accuracy by providing more information on the evolutionary rate and transmission rate of the pathogen.

Throughout our work we assumed neutrality, but in some cases selection can lead to phylogenetic and transmission inference biases [37]. When information on selected sites is available, removing them might alleviate these biases; alternatively, models of site variation in substitution rates (which are available in BEAST2 and can therefore be used with SCOTTI) can alleviate phylogenetic inference biases in these scenarios.

In conclusion, we have presented a new method to reconstruct transmission events, SCOTTI, that addresses the urgent need for software to analyse genomic and epidemiological data while accommodating for incomplete or patchy host sampling, mixed infections, and within-host variation. For these reasons, our method can help to reconstruct transmission histories in a broad range of outbreaks, both bacterial and viral. This information will in turn be essential for devising effective strategies to fight the spread of infectious disease.

## Software Availability

SCOTTI is distributed as an open source package for the Bayesian phylogenetic software BEAST2. It can be downloaded from https://bitbucket.org/nicofmay/scotti/ or via the BEAUti interface [38] of BEAST2.

## Materials and Methods

### Approximate Structured Coalescent Model

Recently, we proposed a BAyesian STructured coalescent Approximation (BASTA) that uses the structured coalescent framework (also known as the coalescent with migration) to infer migration rates and events between populations [27]. BASTA requires substantially less computational time than the exact structured coalescent, particularly when more than just a few populations are considered, by using approximations similarly to [39, 40]. Here, we use the modelling approximations of BASTA in an epidemiological setting, where we model each host as a distinct pathogen population and transmissions as migration events (see Fig 2B). A list of the symbols used hereafter is given in Table A in S1 Text.

To allow the inclusion of epidemiological data, each population (host) *d* ∈ *D* is associated with an exposure interval limited by an introduction time *d*_*i*_ ∈ (−∞, +∞] and a removal time *d*_*r*_ ∈ [−∞, +∞), with *d*_*r*_ < *d*_*i*_ (we consider time backward as typical in coalescent theory). The interval [*d*_*r*_, *d*_*i*_] represents the exposure interval for population *d*, outside of which *d* cannot host any pathogen lineage. *d*_*i*_ and *d*_*r*_ represent respectively the times at which first it was possible for the host to have been infected, and last to have been infectious. For example, in a nosocomial outbreak, *d*_*i*_ and *d*_*r*_ would represent respectively the time of arrival and departure of host *d* into and from the infected hospital or ward. We assume that *d*_*i*_ and *d*_*r*_ are provided by the user and are therefore hereby treated as auxiliary data (we do not model host exposure, and exposure times are always conditioned on). In the worst case scenario where no information on host *d* exposure is provided, it is assumed that *d* is exposed for the whole outbreak (*d*_*i*_ = +∞ and *d*_*r*_ = −∞). We will denote as ***E*** the collection of exposure times. The number of populations *n*_*D*_ is not fixed, but is estimated within a range specified by the user. In the remainder of this work, we will assume that non-sampled demes have unlimited exposure times, but we also provide the option in SCOTTI of specifying regularly distributed introduction and removal times. Here, *n*_*D*_ does not necessarily correspond to the number of non-sampled intermediate hosts in the outbreak, as each host can be infected multiple times, so a non-sampled host due to its infinite exposure time can model more than real life one non-sampled intermediate host. An additional important difference to [27] is that we assume that the migration (or infection) rate *m* is the same between each pair of hosts for the time that they are both exposed. Also, all demes are assumed to have the same effective population size *N*_*e*_. This means that we assume that transmission is *a priori* equally likely between any pair of exposed hosts, and that all hosts have equal, and constant, within-host pathogen evolution dynamics. These assumptions of equal population sizes and migration rates simplify the model and distinguish it from classical structured coalescent methods. In fact, rather than focusing on estimating differences in migration rates and population sizes as in typical structured coalescent methods, we focus on the inference of migration (that is, transmission) events. Yet, these assumptions could be relaxed for example to account for geographically structured hosts or for known contact network. For the time that a host *d* is not exposed, migration rate into or out of *d* is 0.

We assume that a set of samples *I* is provided, where each sample *i* ∈ *I* comes with an aligned sequence *s*_*i*_ ∈ *S*, a sampling date *t*_*i*_ ∈ *t*_*I*_, and a sampled host *l*_*i*_ ∈ *L*. We allow any number of samples from any host, including none (for non-sampled hosts). In this study, we assume that the molecular evolution process follows a time-homogeneous and site-homogeneous HKY model [41], with parameters ***μ***. However, it could be as general as allowed by BEAST2. We denote *T* the bifurcating tree that elucidates the phylogenetic relationships of the samples, and *M* the migration history of all lineages (the collection of all migration/transmission events).

To infer the transmission history in a Bayesian statistical framework, we aim to approximate the following joint posterior distribution:

$$P\left(T,M,n_{D},\mu ,m,N_{e},|S,t_{I},L,E\right)\propto \propto P\left(S|T,t_{I},\mu \right)P\left(T,M|t_{I},L,m,N_{e},E,n_{D}\right)P\left(\mu ,m,N_{e},n_{D}\right).$$ (1)

The first term on the right hand side is the likelihood of the sequences given the genealogy and substitution model. It assumes that sequences evolves down the tree according to a continuous time Markov chain and it can be generally calculated with Felsenstein’s pruning algorithm [42]. The second term is the probability density of the genealogy and migration history. The third term represents the joint prior distribution on the parameters of the nucleotide substitution model and the migration model. Generally, exploring the space of all possible migration histories is computationally demanding even for moderate numbers of populations [27]. For this reason, we integrate over all migration histories by approximating the following posterior distribution as in BASTA [27]:

$$P\left(T,n_{D},\mu ,m,N_{e}|S,t_{I},L,E\right)\propto P\left(S|T,t_{I},\mu \right)P\left(T|t_{I},L,m,N_{e},E,n_{D}\right)P\left(\mu ,m,N_{e},n_{D}\right).$$ (2)

To approximate *P*(*T*|*t*_*I*_, *L*, *m*, *N*_*e*_, ***E***, *n*_*D*_) in Eq 2, we consider the probability density of each time interval between successive events (coalescence, sampling, population introduction, or population removal events). The steps below are very similar to those in BASTA, the main differences being that here we assume equal migration rates and population sizes, and we account for population introductions and removals. Denoting each interval *A*_*i*_ = [*α*_*i*−1_, *α*_*i*_], where *α*_*i*_ is the older event time of *A*_*i*_ and *α*_*i*−1_ the more recent, the probability density of interval *A*_*i*_ can be written as

$$L_{i}=\exp\left[-\int_{\alpha_{i-1}}^{\alpha_{i}}\sum_{d\in D}\frac{1}{2}\sum_{l\in \Lambda_{i}}\sum_{l^{\prime }\in \Lambda_{i},l^{\prime }\neq l}P(d_{l}=d,d_{l^{\prime }}=d|t)\frac{1}{N_{e}}dt\right]E_{i},$$ (3)

where Λ_*i*_ is the set of all extant lineages during interval *A*_*i*_, *d*_*l*_ is the host to which lineage *l* belongs, and *P*(*d*_*l*_ = *d*, *d*_*l*′_ = *d*|*t*) is the probability that lineages *l* and *l*′ are in the same host *d* at time *t*. *E*_*i*_ is the contribution of the particular event:

$$E_{i}=\left\{\begin{array}{cc} \sum_{d\in D}P_{l,\alpha_{i},d}P_{l^{\prime },\alpha_{i},d}\frac{1}{N_{e}} & \text{if}\;\text{it}\;\text{is}\;\text{a}\;\text{coalescence}\;\text{between}\;l\;\text{and}\;l^{\prime }\text{,}\\ 1 & \text{otherwise.} \end{array}\right.$$ (4)

To approximate *L*_*i*_ we substitute *P*(*d*_*l*_ = *d*, *d*_*l*′_ = *d*|*t*) with *P*(*d*_*l*_ = *d*|*t*)*P*(*d*_*l*′_ = *d*|*t*), which corresponds to modelling lineages as migrating independently of each other within an interval between events. This is an approximation in general, but as shown in [27] it has limited effect on estimation. As shorthand, we define ***P***_*l*,*t*_ to be the vector whose *d*th element is *P*_*l*,*t*,*d*_ = *P*(*d*_*l*_ = *d*|*t*). Next, we split each interval *A*_*i*_ into two sub-intervals of equal length *A*_*i*1_ = [*α*_*i*−1_, (*α*_*i*_ + *α*_*i*−1_)/2] and *A*_*i*2_ = [(*α*_*i*_ + *α*_*i*−1_)/2, *α*_*i*_], and replace ***P***_*l*,*t*_ with ***P***_*l*,*α*_*i*−1__ for all *t* in *A*_*i*1_ and ***P***_*l*,*α*_*i*__ for all *t* in *A*_*i*2_. This corresponds to approximating the distribution of lineages among hosts within an interval, as the same distribution at the interval boundaries. As the vast majority of intervals are generally relatively short, and no event occurs within them, this approximation has limited effect but substantially reduces the computations as now we only have to calculate ***P***_*l*,*t*_ at interval boundaries. We also call *τ*_*i*_: = *α*_*i*_ − *α*_*i*−1_. The approximated probability density contributions of *A*_*i*1_ and *A*_*i*2_ become:

$${\tilde{L}}_{i1}=\exp\left[-\frac{\tau_{i}}{2}\sum_{d\in D}\frac{1}{2}\sum_{l\in \Lambda_{i}}\sum_{l^{\prime }\in \Lambda_{i},l^{\prime }\neq l}P_{l,\alpha_{i-1},d}P_{l^{\prime },\alpha_{i-1},d}\frac{1}{N_{e}}\right]$$ (5)

and

$${\tilde{L}}_{i2}=\exp\left[-\frac{\tau_{i}}{2}\sum_{d\in D}\frac{1}{2}\sum_{l\in \Lambda_{i}}\sum_{l^{\prime }\in \Lambda_{i},l^{\prime }\neq l}P_{l,\alpha_{i},d}P_{l^{\prime },\alpha_{i},d}\frac{1}{N_{e}}\right]E_{i}^{\prime }.$$ (6)

The probability density of the genealogy under the structured coalescent, integrated over migration histories, is finally approximated as

$$P\left(T|t_{I},L,m,N_{e},E,n_{D}\right)\approx \prod_{i}{\tilde{L}}_{i1}{\tilde{L}}_{i2}.$$ (7)

The probability distribution of lineages among demes is updated iteratively starting from the most recent event toward the past as

$$P_{l,\alpha_{i},d}=P_{l,\alpha_{i-1},d}\left(\frac{1}{D_{i}}+\frac{D_{i}-1}{D_{i}}e^{-\tau_{i}m}\right)+\left(1-P_{l,\alpha_{i-1},d}\right)\left(\frac{1}{D_{i}}-\frac{1}{D_{i}}e^{-\tau_{i}m}\right)$$ (8)

for any host *d* exposed during the considered interval, where *D*_*i*_ is the number of hosts (sampled or non-sampled) exposed during interval *A*_*i*_. This comes from the assumption that any lineage migrates away from the current host at total rate *m*, and uniformly towards all other extant hosts. For a lineage *l* sampled from deme *d* at time *t*, ***P***_*l*,*t*_ is a vector whose *d*th element equals one and all other entries equal zero. If lineages *l*_1_ and *l*_2_ coalesce to an ancestral lineage *l* at time *t*, then

$$P_{l,t}=\frac{\left(P_{l_{1},t,1}P_{l_{2},t,1},\ldots ,P_{l_{1},t,n_{D}}P_{l_{2},t,n_{D}}\right)}{\sum_{d=1}^{n_{D}}P_{l_{1},t,d}P_{l_{2},t,d}},$$ (9)

which is the normalised entrywise product (element by element product) of the distributions of the coalescing lineages. If instead *α*_*i*_ = *d*_*i*_ is the introduction time for a deme, all remaining lineages in host *d* are forced to migrate out of host *d*. First, ∀*l* ∈ Λ_*i*_ we update the probabilities as in Eq 8. Then, ∀*l* ∈ Λ_*i*_, *P*_*l*,*α*_*i*_,*d*_ is set to 0, and its value is distributed uniformly over all other hosts. If the considered event is a removal of host *d*, Eq 8 is used again, and ∀*l* ∈ Λ_*i*_ *P*_*l*,*α*_*i*_,*d*_ is initiated with the value 0.

Samples from the posterior distribution in Eq 2 are simulated via a Monte Carlo Markov Chain (MCMC). For each of the MCMC samples we simulate hosts at the internal nodes of *T*, and numbers of transmission events along branches, using the same technique as in [27]: first, a host for the root of *T* is sampled according to its posterior probability (Eq 9); then, moving iteratively from the root to the tips, a host is sampled for the remaining internal nodes again according to Eq 9, but also conditional on the host already sampled for the parent node. This procedure accounts for the dependencies among sampled hosts at different internal nodes. Secondly, the numbers of transmission events on each branch are sampled under a Poisson distribution depending on the migration rate *m* and conditional on if the previously sampled hosts at the extremities of the considered branch are the same or not.

SCOTTI allows a large number of populations to be investigated, as the assumption of uniformity of migration rates and effective population sizes greatly reduces the computational demand and parameter space compared to [27]. Also, all non-sampled hosts with the same exposure interval are de facto identical, so usually *n*_*D*_ has no effect on the computational demand of SCOTTI. Example files and data from the analyses described hereby can be found in S1 Data.

### Simulations of Pathogen Evolution

We test the performance in transmission inference of SCOTTI and Outbreaker using a broad range of simulation scenarios. We simulate within-outbreak pathogen evolution using the transmission events observed in two example real-life outbreaks. For half of simulations we use a subset of the FMDV transmission history inferred in [15] (hereby referred to as “transmission history 1”) including 20 UK farms infected during the 2001 outbreak. For the other half of the simulations we use the HIV transmission history described in [8] (hereby referred to as “transmission history 2”) of an outbreak occurred between 1980 and 1983, where a male contracted HIV in 1980 and spread it to six females who subsequently infected two male sexual partners and two children. A description of the transmission history of both scenarios is depicted in Fig C in S1 Text.

While we simulate the coalescent process randomly, the transmission process is fixed *a priori (but see further simulation settings used below)*, so that always the same transmission events at the same time are considered, and only within-host evolution of lineages is varied in different replicates. We use a variant of the multi-species coalescent [31] which is similar to the model used recently in comparable simulations [12, 24]. The model used for simulations is considerably different to both the SCOTTI and Outbreaker models of pathogen evolution (see Fig 2). Hereby each host, during its time of infection, is modelled as a pathogen population with constant effective population size *N*_*e*_, which is the same for all hosts. Lineages within a host can freely coalesce back in time as in the standard coalescent.

In addition to within-host evolution, we want to simulate a typical transmission: a small proportion of the pathogen population passed on at transmission (due to limited inoculum size), followed by rapid growth in the recipient (see e.g. [43, 44]). Therefore, we simulate transmission as a backward in time instantaneous bottleneck in the recipient, followed back in time by the merge of the donor and recipient populations into the donor host. The bottlenecks simulated can have two effect sizes: either equivalent to the drift of *N*_*e*_ generations (a weak bottleneck through which two lineages have a probability of ≈ 63% of coalescing), or 100*N*_*e*_ generations (a strong bottleneck through which two lineages almost surely coalesce). For half of the simulation scenarios we use a weak bottleneck, for the other half a strong one. In the population merger after the bottleneck all lineages remaining in the recipient host are moved to the donor host. Transmission bottlenecks are neither modelled in SCOTTI, nor in Outbreaker (Fig 2).

Finally, half of the simulations are performed providing one sample per host, the other half providing two samples per host, although Outbreaker is only used with one sample per host as it is the only permitted scenario. In summary we have 2 × 2 × 2 = 8 groups of simulations:

- Weak vs strong bottleneck
- First vs second transmission history
- One vs two samples per host.

For each of the aforementioned eight groups, eight different scenarios (or subgroups) are simulated, for a total of 64 distinct simulation settings. We define a basic subgroup (called “base”), and seven variants, in each of which one aspect of the base subgroup is modified. In “base”, sampling times are picked uniformly at random and independently within host exposure times, the average time of infection is 2*N*_*e*_ generations, host is sampled, the alignment length is 1500 bp, and the epidemiological data provided to SCOTTI is accurate (introduction and removal times correspond to infection and recovery time of hosts). The seven variant settings are:

- **Long infection**—the intervals of infection are five times longer (on average 10*N*_*e*_ generations).
- **Abundant genetic**—the alignment is 10 times longer (15000 bp).
- **Early sampling**—samples are collected very early in infection, that is, 5% of the total infection time after infection.
- **Late sampling**—samples are collected at recovery time for each host.
- **Few missing**—one host in the outbreak is not sampled (host O for transmission history 1 and host P5 for transmission history 2).
- **Many missing**—three hosts are not sampled (O, G, and H for transmission history 1, and P1, P5, and P8 for transmission history 2).
- **Inaccurate epi**—SCOTTI is provided with an exposure interval that is broader than the interval of infection of each host. In particular, if ${\bar{L}}_{i}$ is the average length of infection among hosts, then introduction and removal times are respectively ${\bar{L}}_{i}/2$ earlier than infection and ${\bar{L}}_{i}/2$ later than recovery time.

For each of the total 64 subgroups, 100 datasets are simulated under an HKY substitution model [41] with *κ* = 3, 10^−3^ substitution rate per base per *N*_*e*_ generations, and uniform nucleotide frequencies.

For each simulated dataset we infer transmission with Outbreaker under the HKY substitution model and with 10^6^ MCMC iterations. Each analysis is initiated with a random starting tree and with uniform prior infection and sampling probabilities over the maximum observed infection time interval. We run SCOTTI with an HKY substitution model, between 0 and 2 non-sampled hosts, and 10^6^ MCMC iterations. For both methods we assess the performance by checking how often the correct origin of infection of each sampled host is recovered, and with what posterior probability. If transmission from host 1 to host 2 is inferred (either by SCOTTI or Outbreaker), then the infection origin of host 2 is inferred to be host 1. If an indirect transmission to host 2 is inferred, or host 2 is inferred to be an index host or an imported host, then the infection origin of host 2 is inferred to be non-sampled. Lastly, if SCOTTI infers multiple origins of the same host, then, if more than one origin is a sampled host, we always consider the inference as wrong; otherwise we consider the only sampled origin. We use two metrics to define the accuracy of an origin inference: (i) the number of replicates in which the simulated origin is the one with the highest posterior probability (ii) the average posterior probability of the simulated origin across replicates.

#### Additional simulations with random transmission trees

The simulations outlined above were performed under two fixed transmission trees. This gives us the possibility to investigate which particular transmission events are more accurately reconstructed than others. However, this also restricted us to a very limited number of scenarios. To address the question of how SCOTTI performs under more general and random transmission trees, we simulated outbreaks within a hospital setting. Every outbreak started with one case within the hospital, and two other non-infected patients. Each day, a new non-infected patient was allowed to enter the hospital with 10% probability (while we use days as time units, a different time unit would not qualitatively change the structure of the simulated outbreaks). These new patients remained in the hospital for a sojourn time normally distributed with mean 60 days and standard deviation 10. Sojourn time was therefore pre-determined and not affected by transmission events. Also, patients did not recover from infection. Each day, each infected patient had a 1/60 probability of infecting each other non-infected patient. Each patient was only allowed to be infected once. In the case where patient infectivity was variable, an infectivity multiplier was randomly sampled for each patient with a uniform distribution between 0.5 and 1.5. Infection was halted when a total of 12 patients were infected. A number of infected patients, between 12 and 3 depending on the particular scenario, was randomly selected and sampled. Sampling times were uniformly selected within the second and third quarters of the infected sojourn time for each sampled patient. In the base scenario, each infection lasted 5*N*_*e*_ pathogen generations, and transmission bottlenecks corresponded to 5*N*_*e*_ pathogen generations, causing lineages to coalesce within hosts with very high probability. However, when exploring the effect of within-host diversity, we set the duration of infections and the intensity of the bottleneck to 2,1, 0.5, or 0.2*N*_*e*_ pathogen generations. In the scenario with variable *N*_*e*_, for each patient we sampled a within-host effective population size multiplier from an exponential distribution with mean 1.

#### Simulations for testing computational demands

To test the computational requirements of SCOTTI (Fig K in S1 Text) we performed a set of simulations similar to the above ones. We simulated a certain number of outbreak generations (3,5 or 7), each having a certain number of infected hosts (3,5 or 7). The outbreak starts from an index case that infects all hosts in the first generation. Then the infector of a host in generation *n* > 1 is picked at random from hosts in generation *n* − 1. We also included 1 or 2 samples per host. Bottleneck sizes were fixed to 100*N*_*e*_, and all other details were identical to the “Long infection” scenario in previous simulations. Estimations were run with SCOTTI with 4 million MCMC steps, 4 replicates for each scenario, 10% burn-in and a step size of 50. All replicates reached convergence (ESS>500 for posterior probability).

### Foot and Mouth Disease Virus and *K. Pneumoniae* Data

We apply and compare SCOTTI and Outbreaker on two real datasets: one from the 2007 FMDV outbreak in UK [29] and one from a *K. pneumoniae* outbreak in a Nepali hospital in 2011-2012 [30]. In both cases we use alignments, sampling dates and exposure times as provided in the respective studies. All hosts have a single sample, except IP1b (two samples) for FMDV and PMK4, PMK5, PMK13, PMK26 (two samples) and PMK21 (five samples) for *K. pneumoniae*. When multiple samples are present for the same host, only the oldest sample is provided to Outbreaker (which only allows one sample per host). Outbreaker is run for 2 × 10^6^ MCMC iterations on FMDV, and 10^9^ iterations on *K. pneumoniae*, with initial transmission tree inferred with SeqTrack, an HKY substitution model, and prior infectivity and sampling distributions uniform over the maximum exposure interval observed. SCOTTI is run for 10^8^ MCMC iterations under an HKY substitution model and with between zero and two non-sampled hosts. In all cases we checked MCMC convergence with the likelihood trace and effective sample size. These datasets are provided in S1 Data, and can also be downloaded from https://bitbucket.org/nicofmay/scotti/. From the same link, SCOTTI source files and executables can be freely downloaded.

## Supporting Information

S1 Text Supplementary Text. The supplementary text contains further details of the methods and analyses, in particular all supplementary figures and tables.
(PDF)

S1 Data Supplementary Data. File containing information to replicate results.
(ZIP)

## References

[1] Didelot X, Bowden R, Wilson DJ, Peto TE, Crook DW. Transforming clinical microbiology with bacterial genome sequencing. Nature Reviews Genetics. 2012;13(9):601–612. 10.1038/nrg3226 22868263 PMC5049685

[2] Wilson DJ. Insights from genomics into bacterial pathogen populations. PLoS Pathog. 2012;8(9):e1002874 10.1371/journal.ppat.1002874 22969423 PMC3435253

[3] Köser CU, Ellington MJ, Cartwright E, Gillespie SH, Brown NM, Farrington M, et al Routine use of microbial whole genome sequencing in diagnostic and public health microbiology. PLoS Pathog. 2012;8(8):e1002824 10.1371/journal.ppat.1002824 22876174 PMC3410874

[4] Le V, Diep BA. Selected insights from application of whole-genome sequencing for outbreak investigations. Current opinion in critical care. 2013;19(5):432–439. 10.1097/MCC.0b013e3283636b8c 23856896 PMC4104273

[5] Eyre DW, Cule ML, Wilson DJ, Griffiths D, Vaughan A, O’Connor L, et al Diverse sources of C. difficile infection identified on whole-genome sequencing. New England Journal of Medicine. 2013;369(13):1195–1205. 10.1056/NEJMoa1216064 24066741 PMC3868928

[6] Walker TM, Ip CL, Harrell RH, Evans JT, Kapatai G, Dedicoat MJ, et al Whole-genome sequencing to delineate Mycobacterium tuberculosis outbreaks: a retrospective observational study. The Lancet infectious diseases. 2013;13(2):137–146. 10.1016/S1473-3099(12)70277-3 23158499 PMC3556524

[7] Walker TM, Lalor MK, Broda A, Ortega LS, Morgan M, Parker L, et al Assessment of Mycobacterium tuberculosis transmission in Oxfordshire, UK, 2007–12, with whole pathogen genome sequences: an observational study. The Lancet Respiratory Medicine. 2014;2(4):285–292. 10.1016/S2213-2600(14)70027-X 24717625 PMC4571080

[8] Leitner T, Escanilla D, Franzen C, Uhlen M, Albert J. Accurate reconstruction of a known HIV-1 transmission history by phylogenetic tree analysis. Proceedings of the National Academy of Sciences. 1996;93(20):10864–10869. 10.1073/pnas.93.20.10864 PMC38248 8855273

[9] Harris SR, Feil EJ, Holden MT, Quail MA, Nickerson EK, Chantratita N, et al Evolution of MRSA during hospital transmission and intercontinental spread. Science. 2010;327(5964):469–474. 10.1126/science.1182395 20093474 PMC2821690

[10] Pybus OG, Rambaut A. Evolutionary analysis of the dynamics of viral infectious disease. Nature Reviews Genetics. 2009;10(8):540–550. 10.1038/nrg2583 19564871 PMC7097015

[11] Worby CJ, Lipsitch M, Hanage WP. Within-host bacterial diversity hinders accurate reconstruction of transmission networks from genomic distance data. PLoS Comput Biol. 2014;10:e1003549 10.1371/journal.pcbi.1003549 24675511 PMC3967931

[12] Romero-Severson E, Skar H, Bulla I, Albert J, Leitner T. Timing and order of transmission events is not directly reflected in a pathogen phylogeny. Molecular biology and evolution. 2014;31(9):2472–2482. 10.1093/molbev/msu179 24874208 PMC4137707

[13] Kingman JFC. The coalescent. Stoch Proc Appl. 1982;13(3):235–248. 10.1016/0304-4149(82)90011-4

[14] Volz EM, Frost SD. Inferring the source of transmission with phylogenetic data. PLoS Comput Biol. 2013;. 10.1371/journal.pcbi.1003397 PMC3868546 24367249

[15] Cottam EM, Thébaud G, Wadsworth J, Gloster J, Mansley L, Paton DJ, et al Integrating genetic and epidemiological data to determine transmission pathways of foot-and-mouth disease virus. Proceedings of the Royal Society of London B: Biological Sciences. 2008;275(1637):887–895. 10.1098/rspb.2007.1442 PMC2599933 18230598

[16] Aldrin M, Lyngstad T, Kristoffersen A, Storvik B, Borgan Ø, Jansen P. Modelling the spread of infectious salmon anaemia among salmon farms based on seaway distances between farms and genetic relationships between infectious salmon anaemia virus isolates. Journal of The Royal Society Interface. 2011;8(62):1346–1356. 10.1098/rsif.2010.0737 PMC3140724 21325314

[17] Jombart T, Eggo R, Dodd P, Balloux F. Reconstructing disease outbreaks from genetic data: a graph approach. Heredity. 2011;106(2):383–390. 10.1038/hdy.2010.78 20551981 PMC3183872

[18] Lieberman TD, Michel JB, Aingaran M, Potter-Bynoe G, Roux D, Davis MR Jr, et al Parallel bacterial evolution within multiple patients identifies candidate pathogenicity genes. Nature genetics. 2011;43(12):1275–1280. 10.1038/ng.997 22081229 PMC3245322

[19] Morelli MJ, Thèbaud G, Chadœuf J, King DP, Haydon DT, Soubeyrand S. A Bayesian Inference Framework to Reconstruct Transmission Trees Using Epidemiological and Genetic Data. PLoS Comput Biol. 2012 11;8(11):e1002768 10.1371/journal.pcbi.1002768 23166481 PMC3499255

[20] Ypma R, Bataille A, Stegeman A, Koch G, Wallinga J, Van Ballegooijen W. Unravelling transmission trees of infectious diseases by combining genetic and epidemiological data. Proceedings of the Royal Society of London B: Biological Sciences. 2012;279(1728):444–450. 10.1098/rspb.2011.0913 PMC3234549 21733899

[21] Ypma RJ, van Ballegooijen WM, Wallinga J. Relating phylogenetic trees to transmission trees of infectious disease outbreaks. Genetics. 2013;195(3):1055–1062. 10.1534/genetics.113.154856 24037268 PMC3813836

[22] Jombart T, Cori A, Didelot X, Cauchemez S, Fraser C, Ferguson N. Bayesian reconstruction of disease outbreaks by combining epidemiologic and genomic data. PLoS computational biology. 2014;10(1). 10.1371/journal.pcbi.1003457 24465202 PMC3900386

[23] Mollentze N, Nel LH, Townsend S, Le Roux K, Hampson K, Haydon DT, et al A Bayesian approach for inferring the dynamics of partially observed endemic infectious diseases from space-time-genetic data. Proceedings of the Royal Society of London B: Biological Sciences. 2014;281(1782):20133251 10.1098/rspb.2013.3251 PMC3973266 24619442

[24] Didelot X, Gardy J, Colijn C. Bayesian inference of infectious disease transmission from whole-genome sequence data. Molecular biology and evolution. 2014;31(7):1869–1879. 10.1093/molbev/msu121 24714079 PMC4069612

[25] Hall M, Woolhouse M, Rambaut A. Epidemic reconstruction in a phylogenetics framework: transmission trees as partitions of the node set. PLoS Comput Biol. 2015;11(12):e1004613 10.1371/journal.pcbi.1004613 26717515 PMC4701012

[26] Romero-Severson EO, Bulla I, Leitner T. Phylogenetically resolving epidemiologic linkage. Proceedings of the National Academy of Sciences. 2016;p. 201522930 10.1073/pnas.1522930113 PMC4791024 26903617

[27] De Maio N, Wu CH, O’Reilly KM, Wilson D. New Routes to Phylogeography: A Bayesian Structured Coalescent Approximation. PLoS Genet. 2015;11(8):e1005421 10.1371/journal.pgen.1005421 26267488 PMC4534465

[28] Bouckaert R, Heled J, Kühnert D, Vaughan T, Wu CH, Xie D, et al BEAST 2: a software platform for Bayesian evolutionary analysis. PLoS Comput Biol. 2014;10(4):e1003537 10.1371/journal.pcbi.1003537 24722319 PMC3985171

[29] Cottam EM, Wadsworth J, Shaw AE, Rowlands RJ, Goatley L, Maan S, et al Transmission pathways of foot-and-mouth disease virus in the United Kingdom in 2007. PLoS Pathog. 2008;4(4):e1000050 10.1371/journal.ppat.1000050 18421380 PMC2277462

[30] Stoesser N, Giess A, Batty E, Sheppard A, Walker A, Wilson D, et al Genome sequencing of an extended series of NDM-producing Klebsiella pneumoniae isolates from neonatal infections in a Nepali hospital characterizes the extent of community-versus hospital-associated transmission in an endemic setting. Antimicrobial agents and chemotherapy. 2014;58(12):7347–7357. 10.1128/AAC.03900-14 25267672 PMC4249533

[31] Rannala B, Yang Z. Bayes estimation of species divergence times and ancestral population sizes using DNA sequences from multiple loci. Genetics. 2003;164(4):1645–1656. 12930768 10.1093/genetics/164.4.1645 PMC1462670

[32] Gordo I, Campos PR. Patterns of genetic variation in populations of infectious agents. BMC evolutionary biology. 2007;7(1):1. 17629913 10.1186/1471-2148-7-116 PMC1949404

[33] Gordo I, Gomes MGM, Reis DG, Campos PR. Genetic diversity in the SIR model of pathogen evolution. PloS one. 2009;4(3):e4876 10.1371/journal.pone.0004876 19287490 PMC2653725

[34] Murcia PR, Baillie GJ, Daly J, Elton D, Jervis C, Mumford JA, et al Intra-and interhost evolutionary dynamics of equine influenza virus. Journal of virology. 2010;84(14):6943–6954. 10.1128/JVI.00112-10 20444896 PMC2898244

[35] Brauer F. Compartmental models in epidemiology In: Mathematical epidemiology. Springer; 2008 p. 19–79.

[36] Campos PR, Gordo I. Pathogen genetic variation in small-world host contact structures. Journal of Statistical Mechanics: Theory and Experiment. 2006;2006(12):L12003 10.1088/1742-5468/2006/12/L12003

[37] Lemey P, Derdelinckx I, Rambaut A, Van Laethem K, Dumont S, Vermeulen S, et al Molecular footprint of drug-selective pressure in a human immunodeficiency virus transmission chain. Journal of virology. 2005;79(18):11981–11989. 10.1128/JVI.79.18.11981-11989.2005 16140774 PMC1212611

[38] Drummond AJ, Suchard MA, Xie D, Rambaut A. Bayesian phylogenetics with BEAUti and the BEAST 1.7. Mol Biol Evol. 2012;29(8):1969–1973. 10.1093/molbev/mss075 22367748 PMC3408070

[39] Volz EM. Complex population dynamics and the coalescent under neutrality. Genetics. 2012;190(1):187–201. 10.1534/genetics.111.134627 22042576 PMC3249372

[40] Rasmussen DA, Volz EM, Koelle K. Phylodynamic Inference for Structured Epidemiological Models. PLoS Comput Biol. 2014;10(4):e1003570 10.1371/journal.pcbi.1003570 24743590 PMC3990497

[41] Hasegawa M, Kishino H, Yano Ta. Dating of the human-ape splitting by a molecular clock of mitochondrial DNA. J Mol Evol. 1985;22(2):160–174. 10.1007/BF02101694 3934395

[42] Felsenstein J. Evolutionary trees from DNA sequences: a maximum likelihood approach. J Mol Evol. 1981;17(6):368–376. 10.1007/BF01734359 7288891

[43] Salazar-Gonzalez JF, Salazar MG, Keele BF, Learn GH, Giorgi EE, Li H, et al Genetic identity, biological phenotype, and evolutionary pathways of transmitted/founder viruses in acute and early HIV-1 infection. The Journal of experimental medicine. 2009;206(6):1273–1289. 10.1084/jem.20090378 19487424 PMC2715054

[44] Bull RA, Luciani F, McElroy K, Gaudieri S, Pham ST, Chopra A, et al Sequential bottlenecks drive viral evolution in early acute hepatitis C virus infection. PLoS Pathog. 2011;7(9):e1002243 10.1371/journal.ppat.1002243 21912520 PMC3164670

