# Supplementary material for: SCOTTI: Efficient Reconstruction of Transmission within Outbreaks with the Structured Coalescent
Source: PLoS Comput Biol. 2016 Sep 28;12(9):e1005130. doi: 10.1371/journal.pcbi.1005130 (PMC5040440; doi:10.1371/journal.pcbi.1005130)
Supplement: S1 Text — The supplementary text contains further details of the methods and analyses, in particular all supplementary figures and tables. (PDF) [file pcbi.1005130.s001.pdf]

SCOTTI: Efficient Reconstruction of Transmission within Outbreaks with the Structured Coalescent

S1 Text

Nicola De Maio[1,2,*], Chieh-Hsi Wu[2], Daniel J Wilson[1,2,3]

1 Institute for Emerging Infections, Oxford Martin School, University of Oxford , Oxford, United Kingdom
2 Nuffield Department of Medicine, University of Oxford, Oxford, United Kingdom
3 Wellcome Trust Centre for Human Genetics, University of Oxford, Oxford, United Kingdom

*** E-mail: nicola.demaio@ndm.ox.ac.uk**

Figure A. Different representations of transmission events and phylogeny. In our manuscript and software, we use several graphical representations to emphasize different aspects of the transmission and evolutionary histories of pathogens A) To jointly show the evolutionary history of pathogen lineages and transmission events, we adopt "nested" trees. Black boxes represent different hosts (here H1, H2, and H3) and their exposure intervals, limited by the top and bottom edges of each box. Transmission between hosts is represented by blue tubes. Red dots are sequence samples, and red lines represent the pathogen phylogeny. B) To focus on the transmission events only, we use "beanbag" trees. Each host is represented as a circle, with arrows from donor to recipient host. C) Standard phylogenetic tree relating the sampled sequences. D) To represent transmission and evolutionary history simultaneously without including epidemiological data, we use "Maypole" trees. The phylogenetic tree representing the evolutionary history of the pathogen is annotated with colours (one colour for each host) representing the host within which the lineage is inferred to have been. Transition from one colour to another represents transmission between hosts. In this supplement, a similar graphical format is used, where the tree represents the transmission tree enriched with epidemiological data.

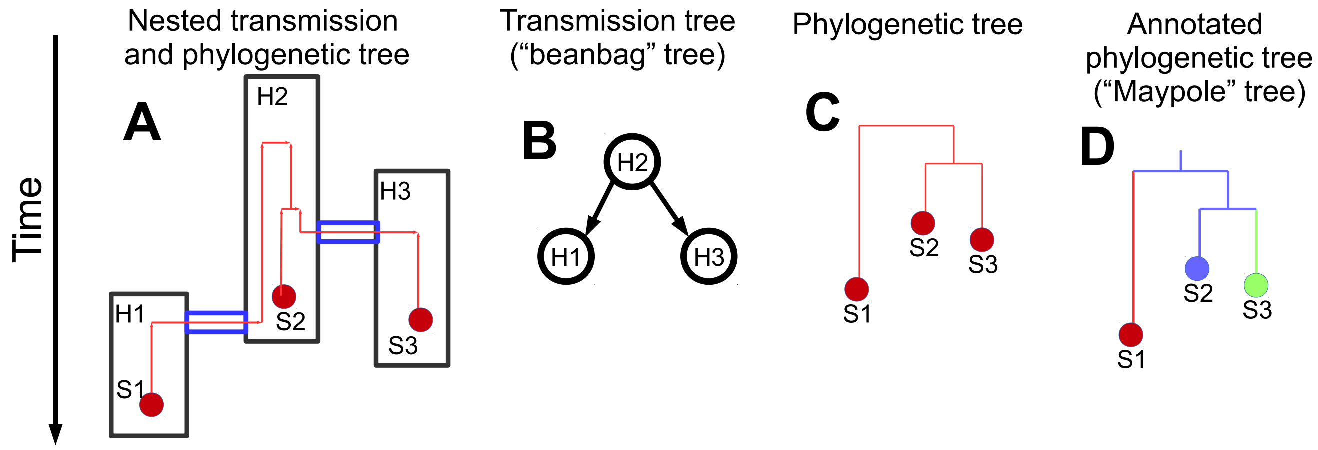

Figure B. Comparison of approaches to transmission inference from genetic data. Several methods to reconstruct transmission from genetic and epidemiological data have been proposed in literature, and here we attempt a comparison and summary of their features. Each row represents a method for inferring transmission history, and each column represents a feature of the model. A red "X" means that the feature is not included, while a green "V" means that the feature is allowed. "–" methods without an explicit phylogenetic structure can indirectly account for phylogenetic uncertainty.

Method	Allows multiple samples from same host	Uses exposure data	Uses sampling times	Uses phylogenetic structure	Accounts for tree uncertainty	Allows non-observed hosts	Allows host distance data	Models within-host evolution	Allows mixed infections	Models partial transmission bottlenecks	Allows compartmentalization model	Infers infection times
Cottam et al 2008	✗	✓	✓	✓	✗	✗	✗	✗	✗	✗	✗	✓
Aldrin et al 2011	✗	✓	✗	✗	-	✗	✓	✗	✗	✗	✗	✓
Ypma et al 2011	✗	✓	✗	✗	-	✗	✓	✗	✗	✗	✗	✓
Jombart et al 2011 (SeqTrack)	✗	✗	✓	✗	-	✓	✓	✗	✗	✗	✗	✗
Morelli et al 2012	✗	✓	✓	✗	-	✗	✓	✗	✗	✗	✗	✓
Ypma et al 2013	✗	✓	✓	✓	✓	✗	✗	✓	✗	✗	✗	✓
Jombart et al 2014 (Outbreaker)	✗	✗	✓	✗	-	✓	✓	✗	✗	✗	✗	✓
Didelot et al 2014	✗	✓	✓	✓	✗	✗	✗	✓	✗	✗	✓	✓
Mollentze et al 2014	✗	✓	✓	✗	-	✓	✓	✗	✗	✗	✗	✓
SCOTTI	✓	✓	✓	✓	✓	✓	✗	✓	✓	✗	✗	✗

Figure C. Accuracy of Reconstructed transmissions in the base simulation scenario. SCOTTI shows overall higher accuracy than Outbreaker in the base simulation setting. Coloured trees represent the (fixed) simulated transmission trees, with one colour associated to each host, and internal nodes corresponding to infection events and times, while tips represent infection clearance times. **A)** transmission history 1, **B)** transmission history 2. In both plots the base simulation setting is considered. The numbers show the accuracy of the infection origin inference for each sampled host. Statistics for each origin are plotted below the branch at which top the respective transmission event occurs. For example, in **A**, statistics regarding the origin of infection of host M are plotted below the branch representing host M. Statistics regarding index hosts (K in **A** and P1 in **B**) are shown next to the root. The origin of infection of a host is defined as either the donor host, if it is sampled, or a general non-sampled origin otherwise. The non-bracketed numbers represent replicates (out of a total of 100) for which the considered origin has been correctly inferred. The numbers in brackets are the average posterior support for the corresponding correct origin over all replicates. For each considered origin, each row represents the results from one of the inference methods ("S1" represents SCOTTI with 1 sample, "S2" SCOTTI with two samples, and "O" Outbreaker). In blue are results for a strong bottleneck (equivalent to the drift of $100\,N_e$ generations), in red for a weak bottleneck (equivalent to the drift of N_e generations).

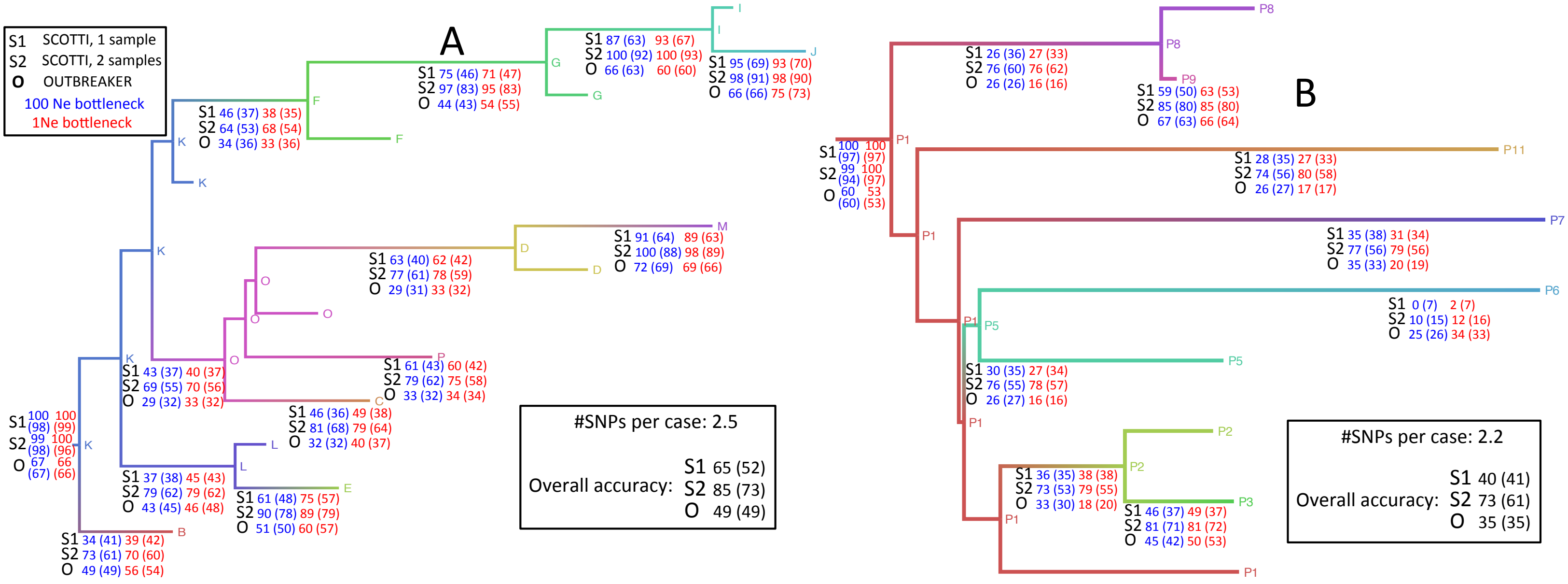

Figure D. Summary of errors in transmission inference. While error in Outbreaker is mostly attributable to the inference of direct transmission between the wrong pair of sampled hosts, in SCOTTI it is more often due to the incorrect attribution of infection source to non-sampled hosts. Pathogen sequence evolution was simulated under transmission history 1, used in **A** and **C**, and transmission history 2, used in **B** and **D**. In **A** and **B** bars represent proportions, expressed as percentages, of incorrect inferences of transmission origin (i.e. donor host) over 100 replicates and all transmission events for each method (differentiated by colour as in legend). The proportion of error due to attribution of transmission to non-sampled hosts is shaded with hashes, while the proportion of error due to attribution to sampled hosts is not shaded. In **C** and **D** bars represent average posterior supports, again expressed as percentages, for the incorrect sources over all patients and replicates. On the X axis are different simulation scenarios.

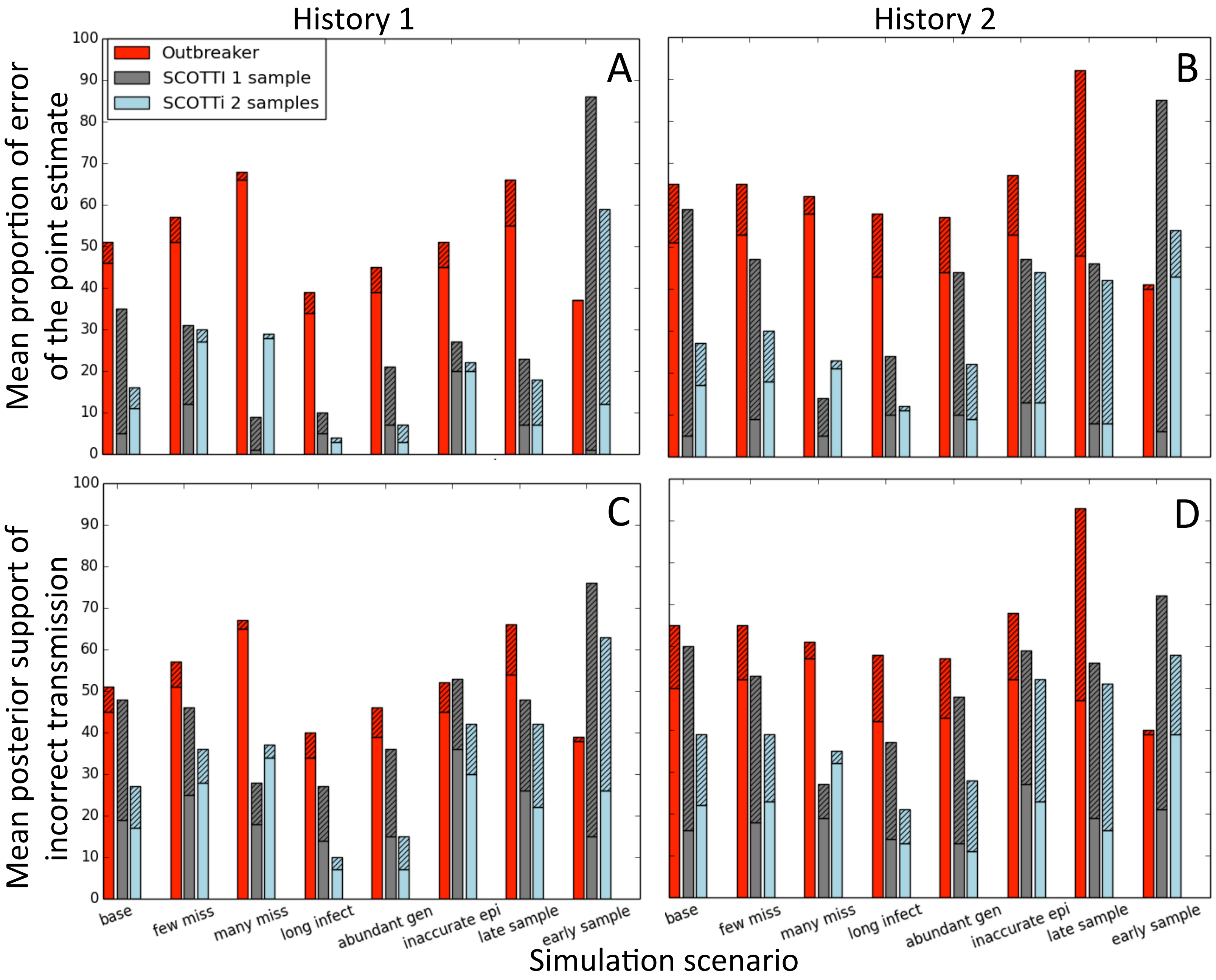

Figure E. SCOTTI and Outbreaker accuracy with non-sampled hosts. SCOTTI shows overall higher accuracy than Outbreaker in the simulation scenarios with some non-sampled hosts. Trees represent simulated transmission trees, internal nodes correspond to infection events and times, and tips represent infection clearance times. The numbers represent inference accuracy as described in Figure C. **A)** Transmission history 1 and one non-sampled host (O). **B)** Transmission history 2 and one non-sampled host (P5). **C)** Transmission history 1 and three non-sampled hosts (O, G, and H). **D)** Transmission history 2 and three non-sampled hosts (P1, P5, and P8).

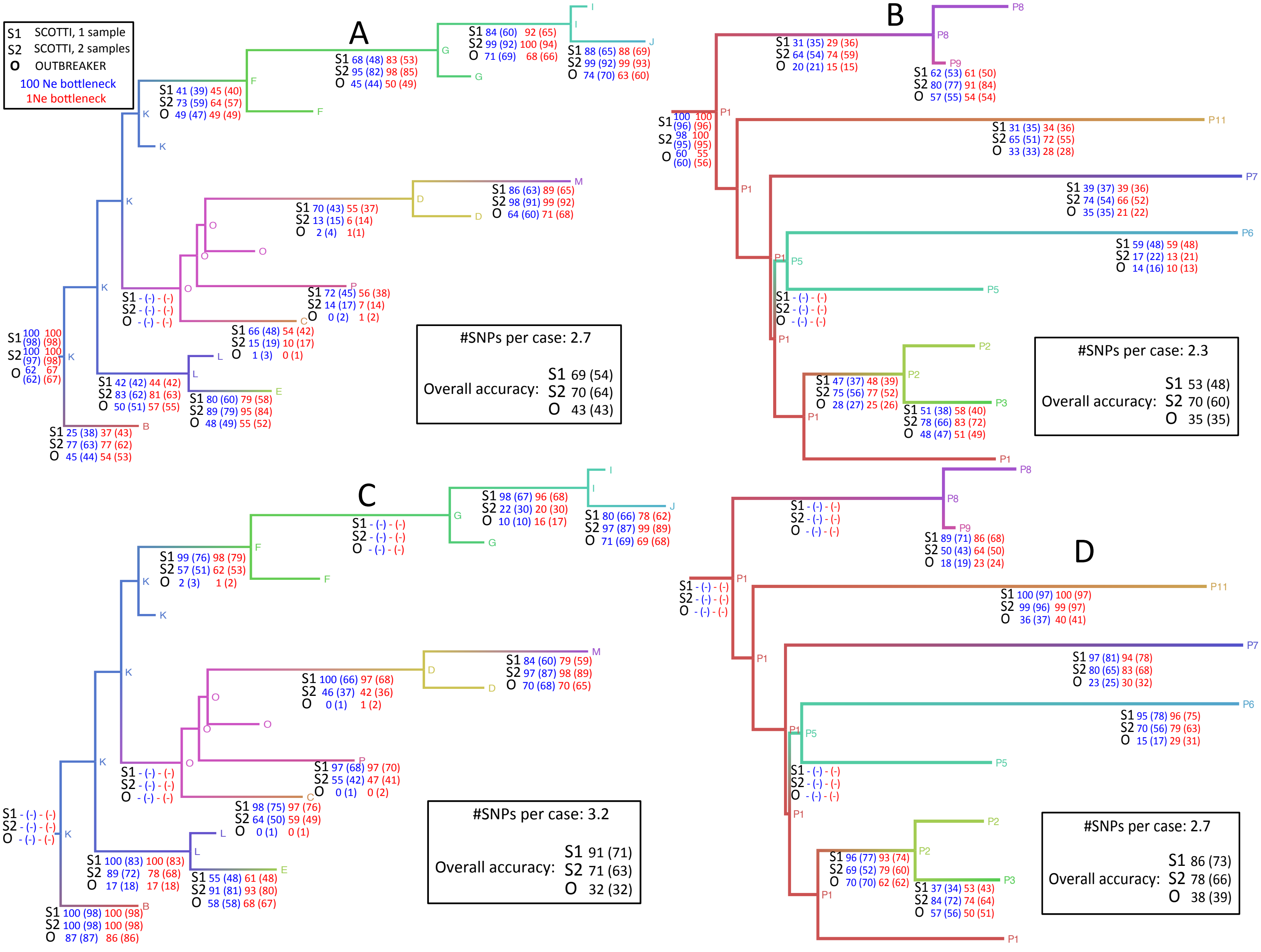

Figure F. Increased accuracy with more genetic variation. Both SCOTTI and Outbreaker show increased accuracy when more genetic variation (and so phylogenetic signal) is provided, and SCOTTI shows overall higher accuracy than Outbreaker. Trees, internal nodes and tips have the same respective meanings as those in Figure E. The numbers represent inference accuracy as described in Figure C. **A)** Transmission history 1 and long infection time (average time of infection 10 N_e generations instead of 2 N_e). **B)** Transmission history 2 and long infection time. **C)** Transmission history 1 and abundant genetic data (15000 base pairs instead of 1500). **D)** Transmission history 2 and abundant genetic data.

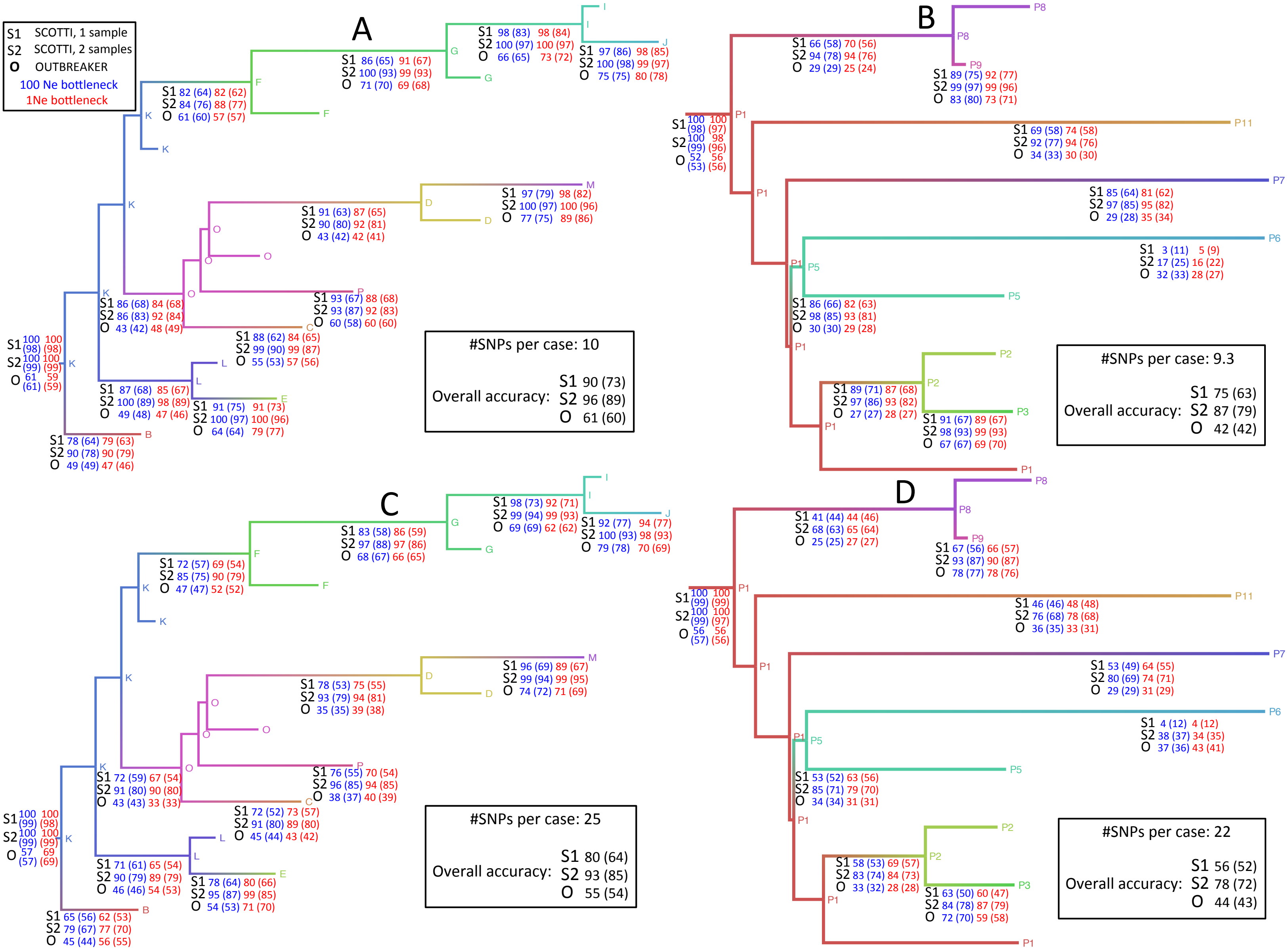

Figure G. Accuracy of host exposure has limited effect on SCOTTI. When provided with inaccurate epidemiological data (exposure intervals are double in length than true ones) SCOTTI is not considerably affected, and still shows higher accuracy than Outbreaker. Trees, internal nodes and tips have the same respective meanings as those in Figure E. The numbers represent inference accuracy as described in Figure C. **A)** Transmission history 1 and inaccurate introduction and removal times (exposure intervals are double in length than the truth). **B)** Transmission history 2 and inaccurate introduction and removal times.

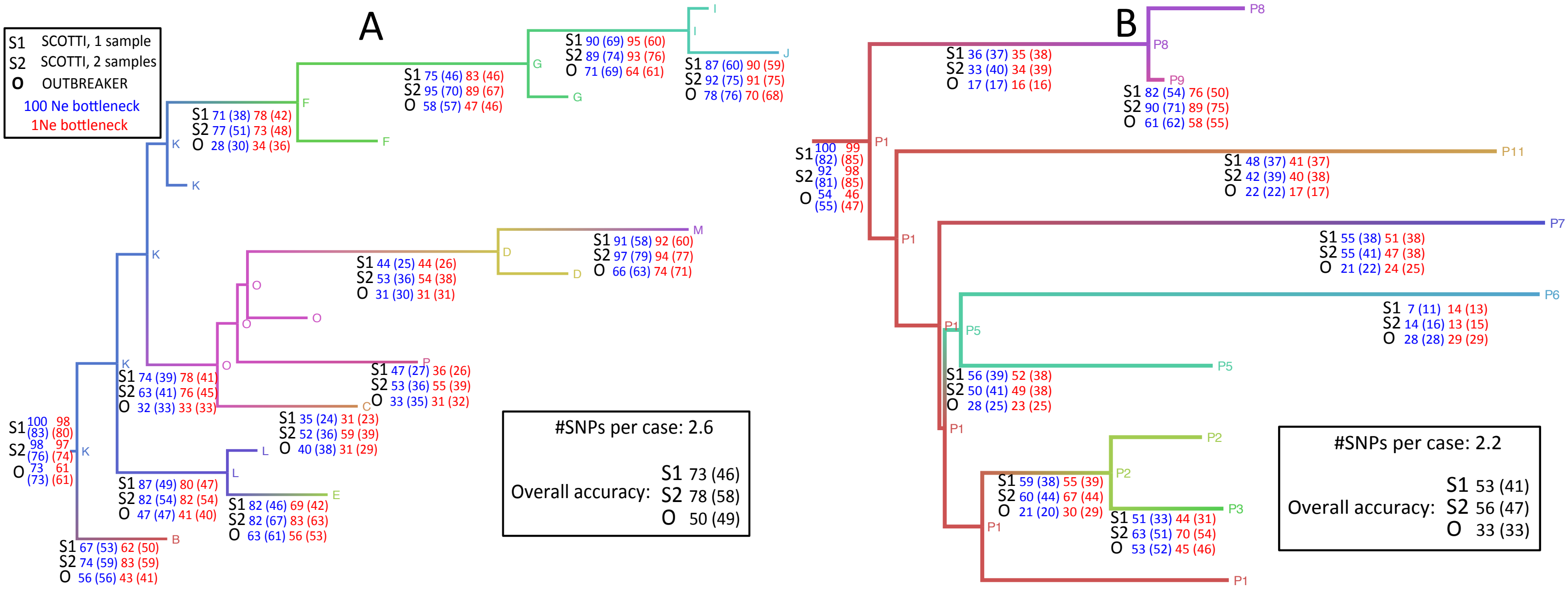

Table A. Symbols used in model description.

D	set of all hosts
n_D	size of D (total number of hosts)
d_r	removal time
d_i	introduction time
E	set of all exposure time information
m	transmission rate
N_e	within-host effective population size
I	set of samples
s_i	genetic sequence of sample i
t_i	time of collection of sample i
l_i	host from which sample i is collected
μ	substitution rate matrix
T	phylogeny relating the samples
M	transmission history
$A_i = [\alpha_{i-1}, \alpha_i]$	time interval between events
$\tau_i = \alpha_i - \alpha_{i-1}$	length of time between events
Λ_i	set of extant lineages at interval i
A_{i1} and A_{i2}	sub-intervals of A_i
D_i	number of hosts exposed at interval i

Figure H. Effects of sampling times on the reconstruction of transmission. With late sampling times (close to infection clearance) SCOTTI shows higher accuracy than Outbreaker, which has high error rates. With samples collected early in infection, instead, SCOTTI has a noticeable decrease in accuracy, and becomes less accurate than Outbreaker. Trees, internal nodes and tips have the same respective meanings as those in Figure E. The numbers represent inference accuracy as described in Figure C. **A)** Transmission history 1 and late sampling (at host clearance). **B)** Transmission history 2 and late sampling. **C)** Transmission history 1 and early sampling (close to infection time). **D)** Transmission history 2 and early sampling.

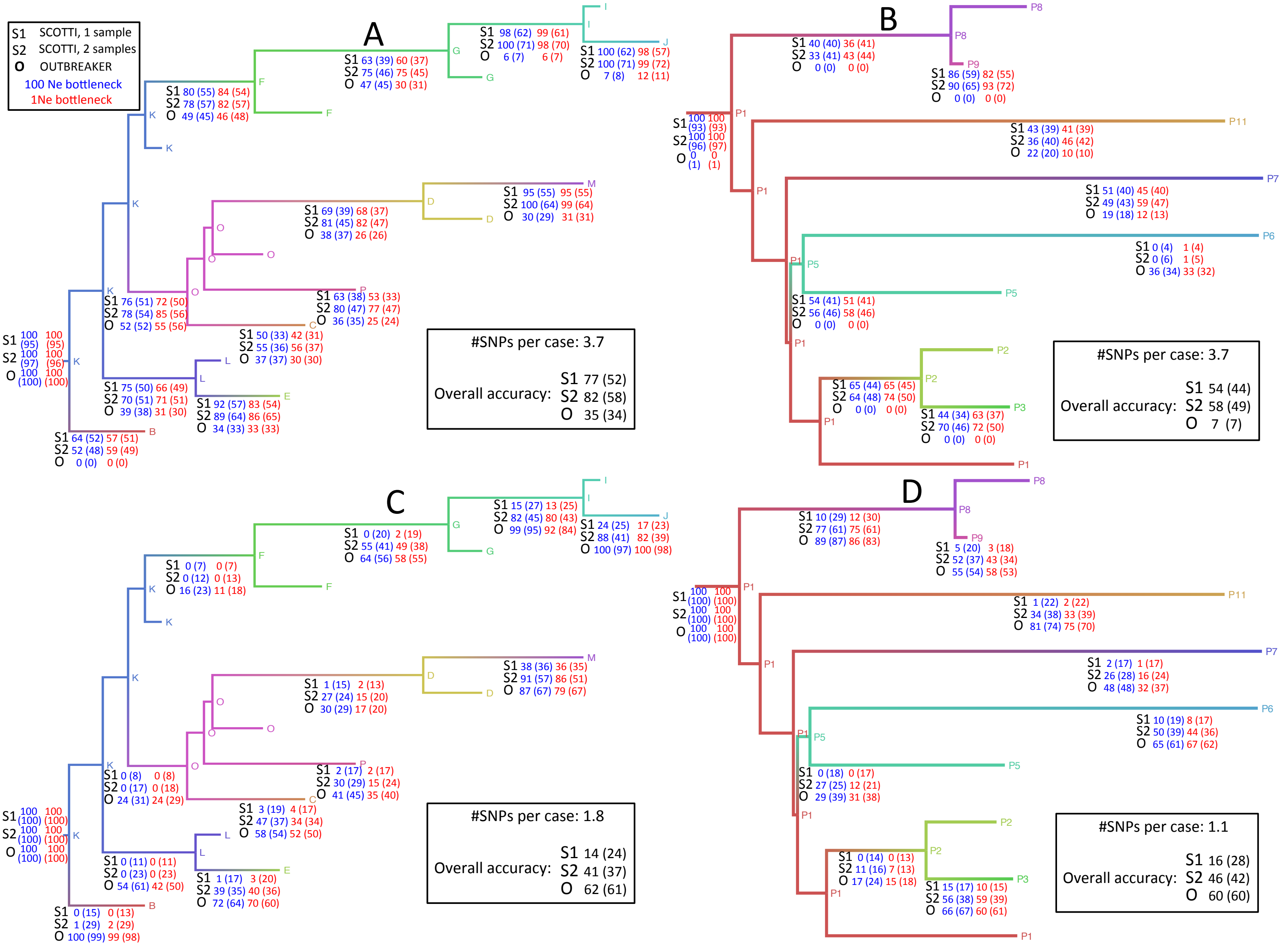

Figure I. Effect of many missing cases and variable host features on SCOTTI. We simulated random transmission histories, 25 for each scenario, and a different sampling and coalescent history for each replicate. On the X axis we have different scenarios, with different numbers of missing cases ("3 miss"-"9miss", out of 12 total cases), different within-host population size for different cases ("variable N_e") and different infectivity for different cases ("variable inf.") Y axis values represent percentages, and estimates are obtained using SCOTTI with one sample per host. **A)** Mean accuracy of the point estimate. **B)** Proportion of times in which the true origin is within the 95% credible set.

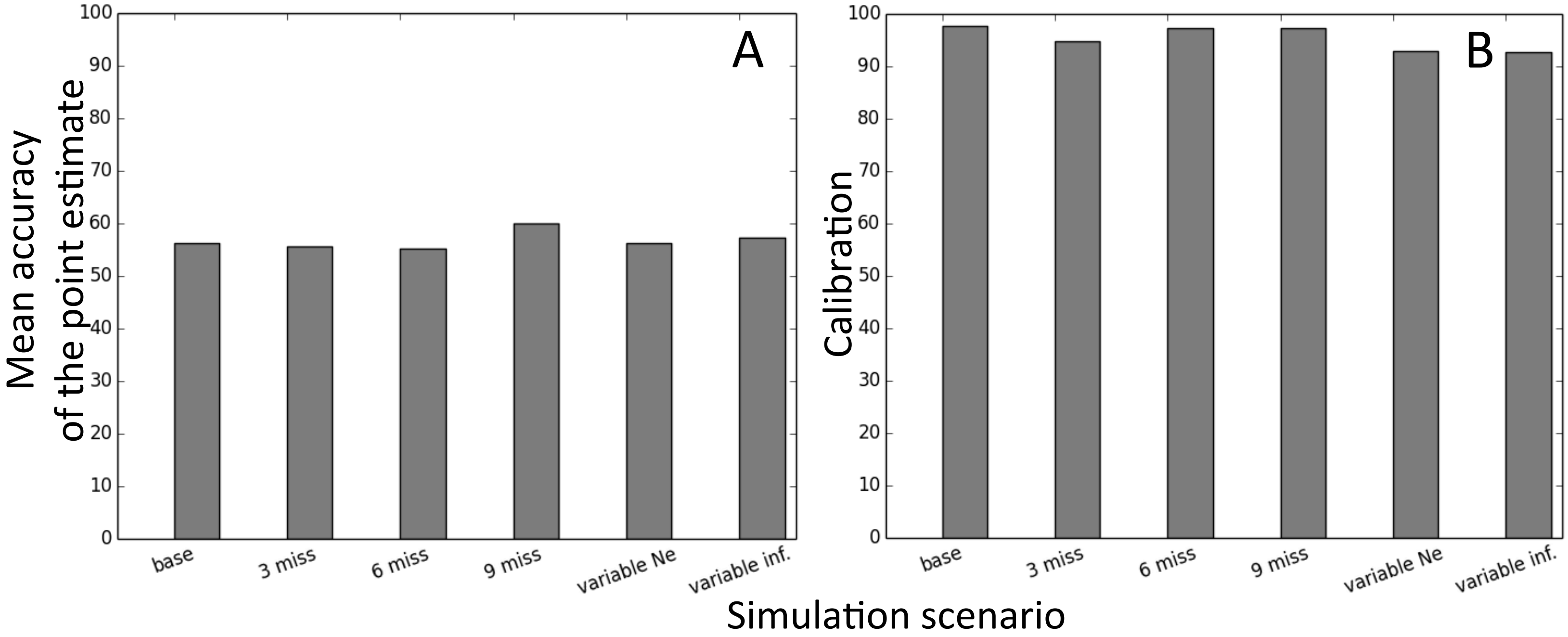

Figure J. Effect of within and between host genetic variation on inference with SCOTTI. We simulated random transmission histories, 25 for each scenario, and a different sampling and coalescent history for each replicate. On the X axis we have different scenarios, with values shown corresponding to the number of N_e pathogen generations for the mean infection length, and also the transmission bottleneck intensity as number of N_e generations. As the value on the X axis decreases, the within-host population size becomes larger, and the transmission bottlenecks become weaker, causing lineages to have lower probabilities of coalescing when they are in the same host. So, on the right end of the plot, within-host diversity is increased with respect to between-host genetic diversity, hindering reconstruction of transmission events. Y axis values represent percentages, and estimates are obtained using SCOTTI with one sample per host (grey bars) or two samples per host (azure bars). **A)** Mean accuracy of the point estimate. **B)** Proportion of times in which the true origin is within the 95% credible set.

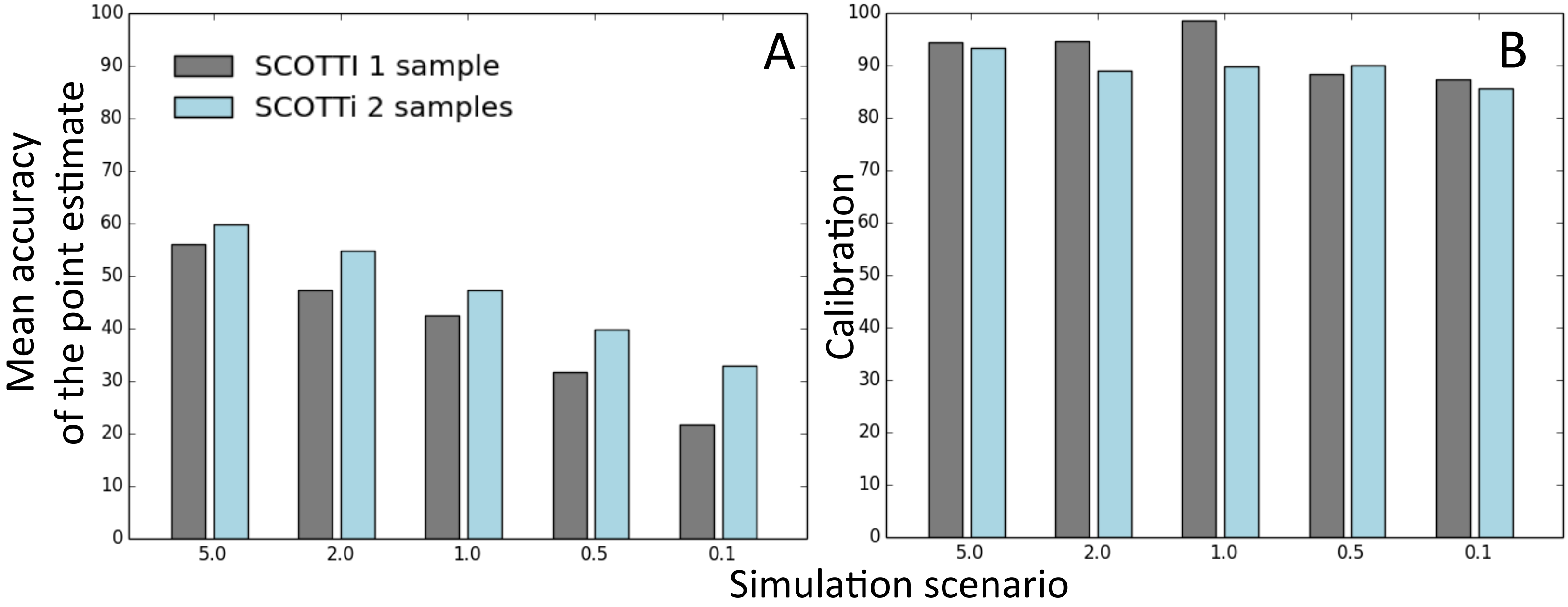

Figure K. Computational demand of SCOTTI. SCOTTI can reconstruct transmission trees for average outbreaks at very limited computational demand. For each combination of number of host generations (3, 5 or 7), number of hosts per generation (3, 5 or 7), and number of samples per host (1 or 2) we performed 4 simulations, with each simulation run abundantly reaching convergence (ESS>200). Here we report the mean runtime (in seconds) for SCOTTI to achieve an ESS of 200 for the posterior probability.

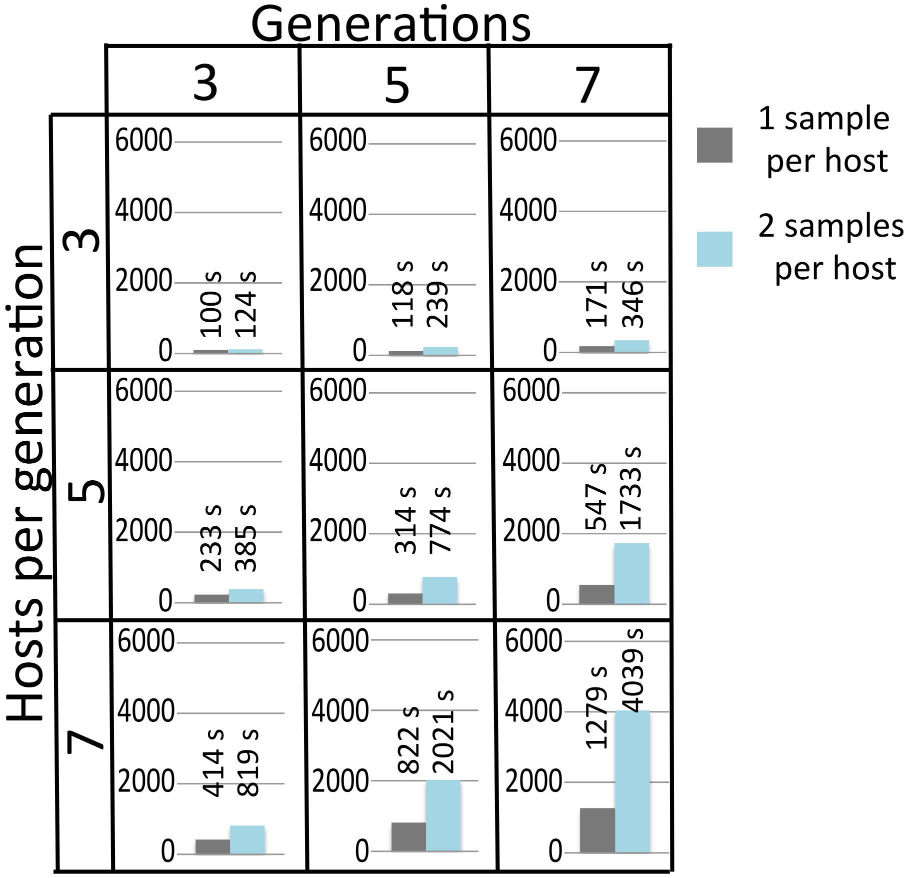

Figure L. Epidemiological and genetic information from the FMDV outbreak [1]. Details of the FMDV outbreak dataset considered. This figure is reproduced from [1]. **A)** Connecting lines represent a nucleotide substitution, thicker lines represent non-synonymous substitutions, with substitutions indicative of adaptation to cell culture coloured green. Sequenced haplotypes (red circles), and putative ancestral virus haplotypes (white circles) are shown. **B)** Lesion age derived infection profiles of holdings overlaid with the outbreak virus geneology. The orange shading estimates the time when animals with lesions were present from the oldest lesion age at post-mortem. For IP2c, there were no clinical signs of disease. The light blue shading represents incubation periods for each holding, estimated to begin no more than 14 days prior to appearance of lesions. The dark blue shading is the infection date based on the most likely incubation time for this strain of 2-5 days. Each UK 2007 outbreak virus haplotype is plotted according to the time the sample was taken from the affected animal (X axis).

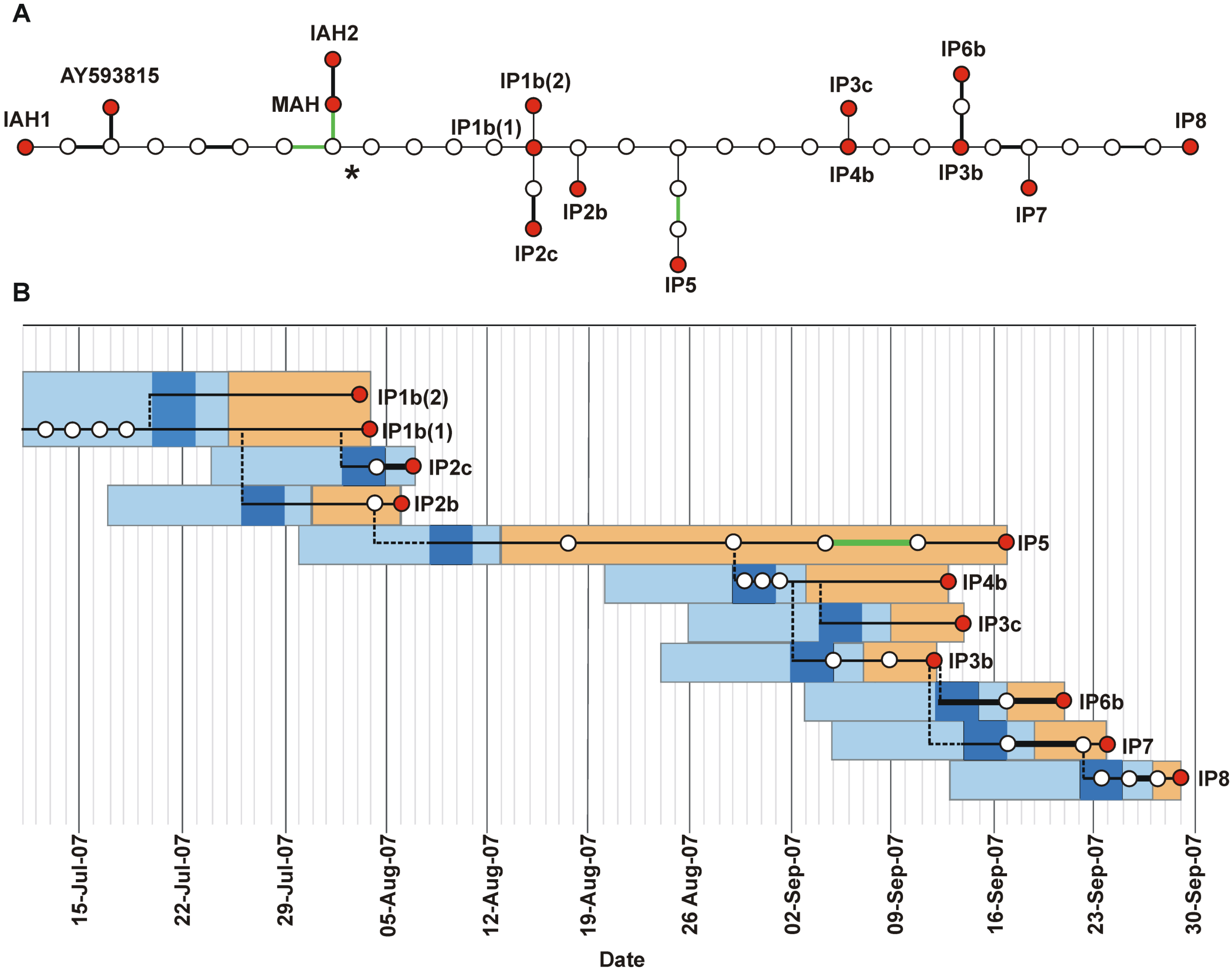

Figure M. Reconstruction of transmission events in a FMDV outbreak using Beastlier [2]. "Beanbag" tree of transmission events inferred with Beastlier from the 2007 South of England FMDV outbreak. Numbers within host circles represent the posterior probabilities of the corresponding host being the index host (the root) of the considered outbreak. Numbers on arrows represent the inferred posterior probabilities of the corresponding direct transmission events. Colour intensity is proportional to posterior probability.

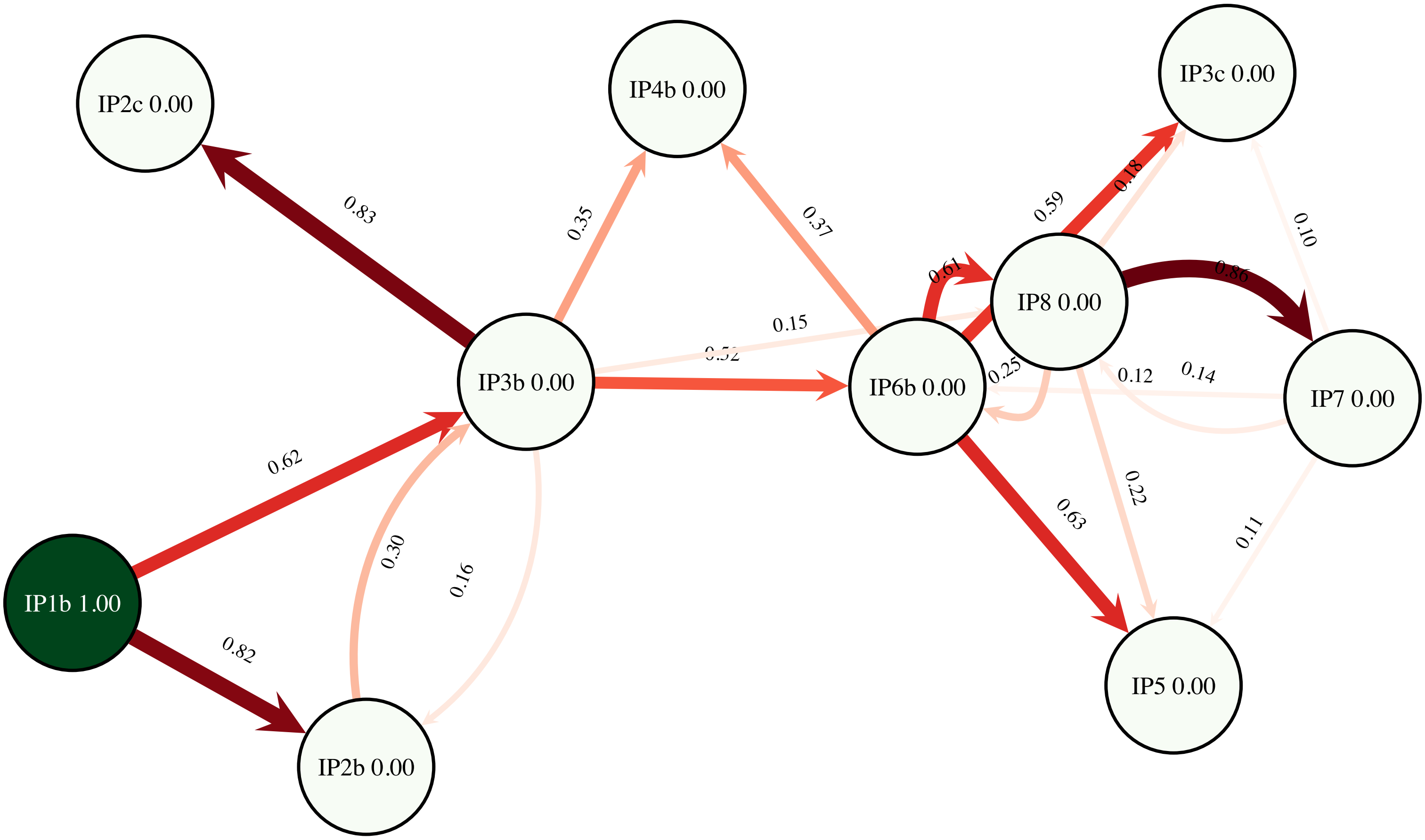

Figure N. Presence of patients within the wards affected by the *K. pneumoniae* outbreak [3]. Timeline of *K. pneumoniae* patients exposures, including individuals who were both part of epidemiologically defined clusters and had genetically linked outbreak strains. This figure is reproduced from [3].

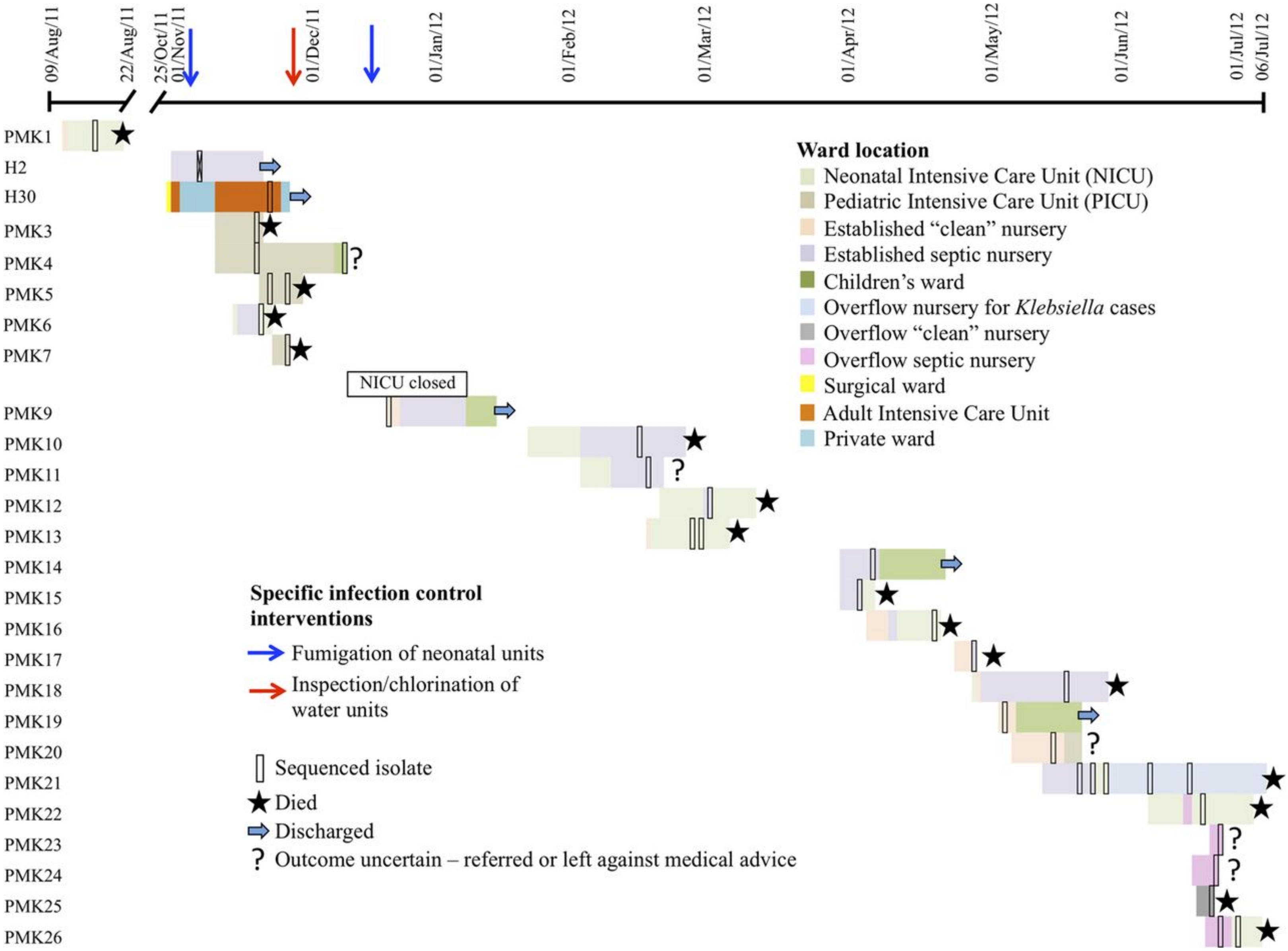

References

1. Cottam EM, Thébaud G, Wadsworth J, Gloster J, Mansley L, Paton DJ, et al. Integrating genetic and epidemiological data to determine transmission pathways of foot-and-mouth disease virus. Proceedings of the Royal Society of London B: Biological Sciences. 2008;275(1637):887–895.

2. Hall M, Woolhouse M, Rambaut A. Epidemic reconstruction in a phylogenetics framework: transmission trees as partitions of the node set. PLoS Comput Biol. 2015;11(12):e1004613.

3. Stoesser N, Giess A, Batty E, Sheppard A, Walker A, Wilson D, et al. Genome sequencing of an extended series of NDM-producing Klebsiella pneumoniae isolates from neonatal infections in a Nepali hospital characterizes the extent of community-versus hospital-associated transmission in an endemic setting. Antimicrobial agents and chemotherapy. 2014;58(12):7347–7357.
